# The Molecular Basis of Kale Domestication: Transcriptional Profiling of Developing Leaves Provides New Insights Into the Evolution of a *Brassica oleracea* Vegetative Morphotype

**DOI:** 10.3389/fpls.2021.637115

**Published:** 2021-03-05

**Authors:** Tatiana Arias, Chad E. Niederhuth, Paula McSteen, J. Chris Pires

**Affiliations:** Bond Life Sciences Center, Division of Biological Sciences, University of Missouri, Columbia, MO, United States

**Keywords:** *Brassica oleracea*, domestication, kale, mustards, plant development, RNA-seq, transcriptomics

## Abstract

Morphotypes of *Brassica oleracea* are the result of a dynamic interaction between genes that regulate the transition between vegetative and reproductive stages and those that regulate leaf morphology and plant architecture. In kales, ornate leaves, extended vegetative phase, and nutritional quality are some of the characters potentially selected by humans during domestication. We used a combination of developmental studies and transcriptomics to understand the vegetative domestication syndrome of kale. To identify candidate genes that are responsible for the evolution of domestic kale, we searched for transcriptome-wide differences among three vegetative *B. oleracea* morphotypes. RNA-seq experiments were used to understand the global pattern of expressed genes during a mixture of stages at one time in kale, cabbage, and the rapid cycling kale line TO1000. We identified gene expression patterns that differ among morphotypes and estimate the contribution of morphotype-specific gene expression that sets kale apart (3958 differentially expressed genes). Differentially expressed genes that regulate the vegetative to reproductive transition were abundant in all morphotypes. Genes involved in leaf morphology, plant architecture, defense, and nutrition were differentially expressed in kale. This allowed us to identify a set of candidate genes we suggest may be important in the kale domestication syndrome. Understanding candidate genes responsible for kale domestication is of importance to ultimately improve Cole crop production.

## Introduction

Domestication is the process of selection used by humans to adapt wild plants to cultivation. During this process, a set of recurrent characters is acquired across a wide diversity of crops (known as the “domestication syndrome”) (Hammer, 1984; Doebley, 1992). Recurrent traits observed in domesticated plants include loss of seed shattering, changes in seed size, loss of photoperiod sensitivity, and changes in plant physiology and architecture (Doebley, 1992; Harlan, 1992; Allaby, 2014). Here, we explore the molecular basis behind the poorly defined vegetative domestication syndrome of kale, a *Brassica oleracea* morphotype, the leaves of which are consumed by humans and are also used as fodder. The domestication syndrome of kale includes apical dominance, ornate leaf patterns, the capacity to delay flower formation and maintain a vegetative state producing a higher yield of the edible portion, nutritional value of leaves, and the ability to defend against herbivores that also give the leaves a unique pungent taste.

The Cole crops (*B. oleracea*) are perennial plants native to Europe and the Mediterranean and include a range of domesticated and wild varieties (Snogerup, 1980; Tsunoda, 1980; Purugganan et al., 2000; Allender et al., 2007; Maggioni et al., 2010). Each crop type is distinguished by domestication traits not commonly found among the wild populations (Maggioni et al., 2010). They include dwarf plants with a main stem that has highly compressed internodes (cabbages), plants with an elongated main stem in which the lateral branches are highly compressed due to very short internodes (Brussels sprouts), plants with proliferation of floral meristems (broccoli) or proliferation of aborted floral meristems (cauliflower), and plants with swollen stems (kohlrabi, Marrow-stem kale) or ornate leaf patterns (kales) (Hodgkin, 1995; Lan and Paterson, 2000). It is not so easy to define which domestication traits are common to all of the above-mentioned types, as opposed to the wild species. At early stages in domestication, plants may have been selected for less bitter tasting, less fibrous, thicker stems, and more succulent storage organs (Maggioni et al., 2010).

The *B. oleracea* genome is derived from an ancient whole-genome triplication event and research has provided evidence that polyploidy and genetic redundancy contribute significantly to the phenotypic variation observed among these crops (Lukens et al., 2004; Paterson, 2005). The genetic basis underlying the phenotypic diversity present in Cole crops has been addressed for only some morphotypes. For example, the cauliflower phenotype of *Arabidopsis* is comparable to inflorescence development in the cauliflower *B. oleracea* morphotype, and it has been shown that the gene *CAULIFLOWER* is responsible for the phenotype observed in both species (Kempin et al., 1995; Carr and Irish, 1997; Purugganan et al., 2000; Smith and King, 2000). However, quantitative trait locus (QTL) mapping for the curd of cauliflower indicates that 86 QTLs are controlling eight curd-related traits, demonstrating this phenotype is under complex genetic control (Lan and Paterson, 2000). Other studies have found and confirmed *BoAP-a1* to be involved in curd formation in cauliflower (Anthony et al., 1995; Carr and Irish, 1997; Gao et al., 2007) and have shown that morphologies in this cultivar are under complex genetic control (Sebastian et al., 2002). The molecular bases of broccoli have also been proposed as involving changes in three codons in the *BoCAL* and *BoAP* genes (Carr and Irish, 1997; Lowman and Purugganan, 1999). A total of five QTLs have been detected for leaf traits in Brussel sprouts, but no significant QTLs have been found for axillary buds (Sebastian et al., 2002). Head formation in cabbage has been suggested as additively inherited (Tanaka et al., 2009). In kale, a recent study suggest that the lobed-leaf trait is quantitatively inherited (Ren et al., 2019).

Definitions of domestication syndrome in kale include traits affecting cultivation, harvesting, and cooking—and its uses—including food, medicines, toxins, and fibers (Denham et al., 2020). Leafy kales are considered the earliest cultivated brassicas, originally used for both livestock feed and human consumption (Maggioni et al., 2010) due to leaves and floral buds with great nutritional value (Velasco et al., 2011). Apical dominance is manifest in kale varieties, involving selection for erect plants with fewer side branches, and more compact plants, which allows more plants to fit into each unit of cultivated soil (Denham et al., 2020).

In kale, leaves are green to purple and do not form a compact head like in cabbage. Crop varieties have surprising variation in leaf size, leaf type, and margin curliness. The over expression phenotype of *knotted1-like homeobox* (*knox*) transcription factors in *Arabidopsis* (Hay and Tsiantis, 2006, 2009; Blein et al., 2008; Kimura et al., 2008) is very reminiscent of the highly lobed margins phenotype of kale, while in other Brassicaceae, a different homeodomain protein is involved in evolutionary changes in leaf complexity at a later stage in leaf development (Vlad et al., 2014). Kale leaf margin characteristics including curliness, blade texture, and tissue overgrowth are some of the more variable morphological characters among varieties but remain to be studied at the molecular level.

Kale leaves also have antioxidative properties and have been shown to reduce cholesterol levels (Velasco et al., 2007; Soengas et al., 2012). Their high content of beta-carotene, vitamin K, vitamin C, and calcium make it one of the most nutritious vegetables available for human consumption (Stewarta and McDougalla, 2012). Kale is a source of two carotenoids: lutein and zeaxanthin (Velasco et al., 2007). Kale leaves also have mustard oils produced from glucosinolates including sulforaphane, a compound suggested to have anticancer properties (Velasco et al., 2007). These natural chemicals most likely contribute to plant defense against pests and diseases and impart a pungent flavor property characteristic of all cruciferous vegetables (Sønderby et al., 2010; Carmona et al., 2011; Hahn et al., 2016).

Here, we used a combination of developmental studies and transcriptomes to understand the vegetative domestication syndrome of kale. Comparisons of kale morphotypes with the “rapid-cycling” morphotype TO1000 and cabbage allow us to identify transcriptome-wide differences that set kale apart. RNA-seq of a mixture of leaf stages for the *B. oleracea* vegetative morphotypes cabbage, kale, and TO1000 reveal gene expression patterns unique to kale and associated developmental and metabolic processes. This allowed us to identify a set of candidate genes we suggest may be important in the kale domestication syndrome.

## Materials and Methods

### Plant Material Used for Microscopy Studies

**FIGURE 1:**
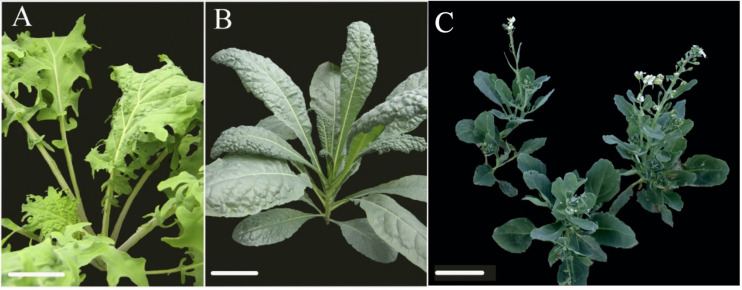
Comparison of mature leaf morphologies within kale varieties and TO1000 used for the developmental studies; red winter kale (*Brassica oleraceae* var. acephala *Red Winter*) was also used in transcriptomics experiments. **(A)** Red winter kale with lobed leaf margins, **(B)** Lacinato Nero Toscana kale (*Brassica oleracea* var. *palmifolia*) with crenulate leaf margins, **(C)** TO1000 (*B. oleracea* var. *alboglabra*) picture provided by Zachary Stansell. Scale bar = 10 cm.

Leaf shape development was recorded for different kale varieties: red winter kale with lobed leaf margins (*B. oleraceae* var. *Red Winter*) and Lacinato Nero Toscana kale (*B. oleracea* var. *palmifolia*) with crenulate leaf margins (Figures 1A,B). Structural features of the meristems and developing leaves were observed during 30 days using environmental scanning electron microscopy (ESEM) and light microscopy (Leica, Frankfurt, Germany). Images were processed with Adobe (San Jose, California, United States) Photoshop, version 7.0. Plants were visually observed and described in the green house until fully grown.

### Plant Material and Experimental Design Used for RNA-Seq Studies

Single accessions of cabbage PI 303629 South Africa (*B. oleracea* var. *capitata*), kale (*B. oleraceae* var. acephala *Red Winter* SI), and the rapid cycling doubled haploid kale-like type HRI TO1000 DH3 (*B. oleracea* var. *alboglabra* from Warwick) (Supplementary Figure 1; Parkin et al., 2014) were grown in an environmental chamber under uniform conditions: 10 h day (light, 7 AM–5 PM; dark, 5:01 PM–6:59 AM daily) and watered every other day. Morphotypes were planted in a completely randomized block design. Initially, two seeds per pot were planted; then, only one was picked (32 plants per morphotype total). A random number generator was used to move plants and flats around every other day.

Tissues were collected 24 days after seeds were planted (cabbage had 2–3 fully expanded leaves, kale had 3–4 fully expanded leaves, and TO1000 had 3–5 fully expanded leaves). The upper portion of the stems, including immature leaves, and apical and lateral meristems were flash frozen in liquid nitrogen. The assumption was made that genes involved in developing different morphologies should be expressed in younger leaves, shoots, and meristems. Three biological replicates per morphotype were collected, each replicate consisting of pooled tissue from eight plants.

### RNA Extraction and cDNA Synthesis

Lysis and homogenization of fresh soft tissue were performed before RNA extraction; PureLink Micro-to-Midi Total RNA Purification System (Invitrogen, Cat. No 12183-018, Carlsbad, California, United States) was used. RNA extraction was done using the Purelink RNA Mini Kit (Ambion, Cat. No. 12183-018A, Carlsbad, California, United States). DNase was not used before proceeding with complementary DNA (cDNA) synthesis as recommended in the protocol. For cDNA synthesis, MINT (Evrogen, Cat. No SK001, Moscow, Russia) was used; this kit enzymes synthesize full-length-enriched double-stranded cDNA from a total polyA^+^ RNA. The cDNA was then amplified by 16–21 cycles of polymerase chain reaction and purified using a PCR Purification Kit (Invitrogen K3100-01 Carlsbad, California, United States).

### Library Construction and Illumina Sequencing

Protocols previously published by Steele et al. (2012) were implemented here. End repair was performed on 400 μl with 3 μg of normalized ds-cDNA prior to ligating barcoding adapters for multiplexing, using NEB Prep kit E600L (New England Biolabs, Ipswich, Massachusetts, United States). Shearing was done using a Bioruptor^®^ sonication device (Diagenode, Denville New Jersey, United States), at 4°C, continuously for 15 min total (7 mix) on high. Three replicates per morphotype were tagged with different adaptors and sequenced on a single lane of an Illumina GAIIx machine.

Oligonucleotides used as adapters were cabbage (1, ACGT; 2, GCTT; 3, TGCT), kale (1, TACT; 2, ATGT; 3, GTAT), and TO1000 (1, CTGT; 2, AGCT; 3, TCAT). To prepare adapters, equal volumes were combined of each adapter at 100 μmol/L floating them in a beaker of boiling water for 30 min and snap cooling them on ice. Ligation products were run on a 2% low-melt agarose gel and size selected for ∼300 bp using an x-tracta Disposable Gel Extraction Tool (Scientific, Ocala Florida, United States). Fragments were enriched using PCR in 50 μl volumes containing 3 μl of ligation product, 20 μl of ddH_2_O, 25 μl master mix (from NEB kit), and 1 μl each of a 25 μmol/L solution of each forward and reverse primer [forward 5’-AAT GAT ACG GCG ACC ACC GAG ATC TAC ACT CTT TCC CTA CAC GAC GCT CTT CCG ATC^∗^ T-3’; reverse 5’-CAA GCA GAA GAC GGC ATA CGA GAT CGG TCT CGG CAT TCC TGC TGA ACC GCT CTT CCG ATC^∗^-3’; both high-performance liquid chromatography (HPLC) purified]. Thermal cycle routine was as follows: 98°C for 30 s, followed by 15 cycles of 98°C for 10 s, 65°C for 30 s, and 72°C for 30 s, with a final extension step of 72°C for 5 min. Products were run on a 2% low-melt agarose gel, and the target product was excised and purified with the tools described above. DNA was purified at each step during the end repair, adapter ligation, size selection, and fragment enrichment with either a Gel Extraction kit (Qiagen, Germantown Maryland, United States) or a DNA QIAquick PCR Purification kit (Qiagen, Germantown Maryland, United States). The three replicates per morphotype were run on one-third of a lane with single-end 98-bp reads on an Illumina GAIIx Genome Analyzer using the Illumina Cluster Generation Kit v2-GA II, Cycle Sequencing Kit v3 (Illumina, San Diego, United States), and image analysis using Illumina RTA 1.4.15.0 (Illumina, San Diego, United States) at University of Missouri DNA Core.

### Data Quality Control and Preprocessing, Alignment, and Differential Expression Analysis

Reads were first trimmed for primer and adapter sequences using Cutadapt v2.4 (Martin et al., 2010) and read quality assessed using FastQC (Andrews, 2010). These were then aligned against the *B. oleracea* TO1000 genome (Parkin et al., 2014) downloaded from EnsemblPlants 44 (Kersey et al., 2018) with two passes of STAR v2.7.1a (Dobin et al., 2013). In the first pass, reads for each sample were aligned for splice-junction discovery, in addition to those junctions provided in the TO1000 annotations. Splice junctions from all samples were combined and provided for a second alignment, after which read counts were calculated for each gene using STAR. Mapping statistics are provided in Supplementary Table 1. Raw sequencing data and unnormalized read counts are publicly available through the Gene Expression Omnibus accession GSE149483.

Read counts were imported into R v3.6.3 (R Core Development Team, 2008), and differential expression was determined using DESeq2 v1.26.0 (Love et al., 2014). Specifically, read counts were normalized, with the TO1000 samples as reference, and fitted to a parametric model. We looked for differentially expressed genes (DEGs) for each pairwise comparison (kale vs. TO1000, kale vs. cabbage, and cabbage vs. TO1000). Comparisons were done to ultimately separate unique kale-morphotype DEGs. Since each pairwise comparison increases the potential number of false positives, these were combined into a single table, and the false discovery rate (FDR) (Benjamini and Hochberg, 1995) was adjusted for the entire table. Genes with an FDR < 0.05 and log-fold change > 1 or < −1 were considered as DEGs. Plots were made using the R packages ggplot2 (Wickham, 2009), pheatmap (Kolde, 2019), and VennDiagram (Chen, 2018). Transcription-associated proteins (TAPs) were identified using the approach described for the construction of PlnTFDB v3.0^1^ (Riaño-Pachón et al., 2007; Pérez-Rodríguez et al., 2010). Scripts for bioinformatic analysis and original figure pdfs and tables are available on GitHub^2^.

### Subgenome Analysis

Assignment of each gene to their respective subgenome were obtained from Parkin et al. (2014). Genes assigned to a subgenome were previously determined by synteny to *Arabidopsis thaliana*. Unassigned genes were considered non-syntenic. Enrichment of DEGs for syntenic vs. non-syntenic and for each subgenome was determined using a Fisher’s exact test (Fisher, 1934) and considered significant with an FDR-corrected *p* < 0.05.

### Gene Annotation, GO, and KEGG Analysis

Gene Ontology (GO) terms (Ashburner et al., 2000) are not currently available for the TO1000 assembly. In order to annotate TO1000 gene models with GO terms, TO1000 protein sequences were Blasted (blastp) against protein sequences from UniProtKB Swiss-Prot (release 2019_05) (UniProt Consortium, 2018) using Diamond (Buchfink et al., 2014), with an *e*-value cutoff of 1e^–5^ and a maximum of one hit per query. UniProt IDs were then used to extract GO terms and mapped to the corresponding TO1000 gene model. In total, 35,422 genes (out of 59,225 total, ∼59.8%) had a GO term assigned. GO term enrichment was performed using topGO v 2.38.1 (Alexa and Rahnenfuhrer, 2019) and GO.db v3.10.0 (Carlson, 2019) with the parent–child algorithm (Grossmann et al., 2007) and Fisher’s exact test. A minimum node size of 5 was required, i.e., each GO term required a minimum of five genes mapping to it in order to be considered. *p*-values were adjusted for FDR and considered significant with an adjusted *p* < 0.05.

KEGG annotations are available for TO1000 (see^3^), but the National Center for Biotechnology Information (NCBI) GeneID was used rather than the gene name in the Ensembl annotations. To address this, a reciprocal Blast (blastp) was performed using Diamond with protein sequences from the Ensemble TO1000 genome and translated coding sequences from NCBI TO1000 genome (accession GCA_000695525.1). NCBI GeneIDs were then assigned to the TO1000 Ensemble genome annotations based on the reciprocal best blast hit. Enrichment of KEGG pathways was then performed in R using clusterProfiler v3.18.0 (Yu et al., 2012), and pathways were considered enriched with an FDR-adjusted *p* < 0.05. Scripts, GO terms, and NCBI GeneID assignments for each gene are available on GitHub (see text footnote 2).

## Results

### Kale Leaf Development

**FIGURE 2:**
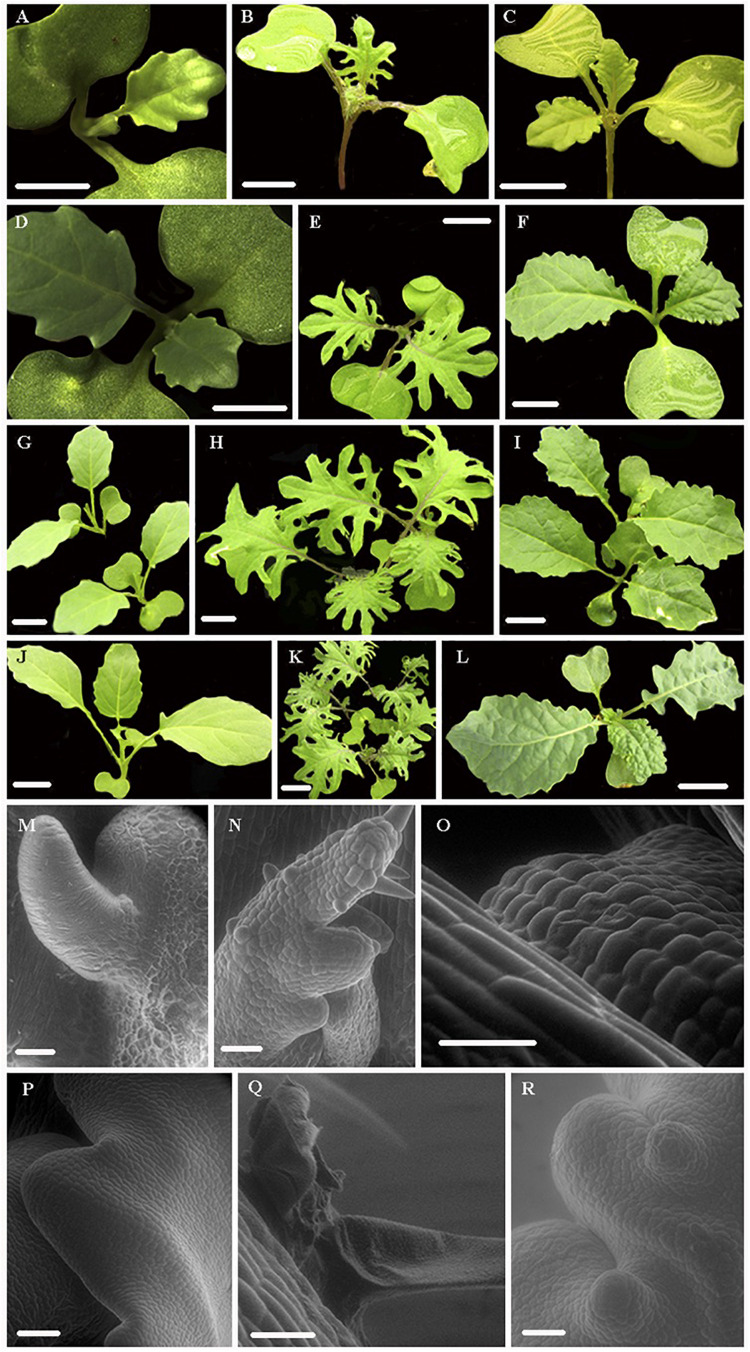
Leaf shape development in two Kale varieties, red winter kale (*Brassica oleraceae* var. *acephala*) and Lacinato Nero Toscana kale (*B. oleracea* var. *palmifolia*) and TO1000 (*B. oleracea* var. *alboglabra*). **(A–L)** Progressive development of the different kale varieties and the rapid cycling TO1000 control (stereoscope and regular pics). Scale bar = 1 cm. **(M–R)** Scanning electron microscopy (SEM) of apical meristem, primordial leaves, and details of lobes in different kale varieties and TO1000 control, scale bar = 5 μm. **(A,D,G,J)** Leaf development series for TO1000. **(B,E,H,K)** Leaf development series for kale with lobed leaf margins (red winter kale). **(C,F,I,L)** Leaf development series for Lacinato Nero Toscana kale with non-lobed leaf margins. **(M,P)** SEM of TO1000 leaf primordia and margin formation detail. **(N)** SEM of red winter kale leaf primordia. **(O)** SEM of Lacinato Nero Toscana kale leaf primordia. **(Q)** SEM of ectopic growth of leaf blade in mature leaves of red winter kale subtended by a trichome. **(R)** Margin formation detail in non-lobed kale (Lacinato Nero Toscana kale).

Developmental transitions and morphological changes were observed in two kale varieties: red winter kale with lobed leaf margins (*B. oleraceae* var. *Red Winter*) and Lacinato Nero Toscana kale (*B. oleracea* var. *palmifolia*) with crenulate leaf margins and the rapid cycling TO1000 DH3 (*B. oleracea* var. *alboglabra* from Warwick) for 22 days (Figures 1, 2). Leaf development encompasses three continuous and overlapping phases. During leaf initiation, the leaf primordium emerges from the flanks of the shoot apical meristem (SAM) at positions determined by specific phyllotactic patterns (Figures 2M,N). In the second phase, the leaf expands laterally, and primary morphogenesis events occur from specific meristematic regions at the leaf margin (blastozones) (Figures 2A–F). In the third phase of secondary morphogenesis, extensive cell expansion and histogenesis occurs (Figures 2G–L,Q,R). A week after planting, all morphotypes had germinated; at first, only the cotyledons were observed. Lobed-kale grew faster than non-lobed kale and TO1000. Using environmental scanning electron microscopy (ESEM), tiny true leaves with lobed margins and abundant trichomes were observed for lobed-kale at 5 days (Figure 2N), while non-lobed kale and TO1000 had not developed lobes or leaves at this point (Figures 2M,O). In 8 day-old lobed kale, the first immature leaves were seen emerging from between the cotyledons and were covered by trichomes (Figure 2B). Ten days after germination, lobed-kale seedlings had abundant trichomes and highly lobed leaf margins; leaf shape was elliptic to obovate and leaves had red venation and red petioles (Figure 2E). In comparison to the kale varieties, TO1000 seedlings had taller hypocotyls and erect stems. Leaf shape was oblanceolate, and leaf margins were sinuate (Figure 2D). Leaf margins in the Lacinato Nero Toscana kale were more sinuate in comparison to TO1000 at this stage (Figure 2F). Twelve day-old lobed-kale produce ectopic meristems in the leaf margins and more sporadically in the leaf blade of mature leaves; microscopic scales surrounded these ectopic meristems (Figure 2Q). Ectopic meristems located at the tip of each lobe had a vascular bundle inserted to them and were always subtended by a trichome. Nineteen-day-old lobed kale and TO1000 had four leaves, the fourth one small; while non-lobed kale has three leaves and the third one small. Lobed-kale leaves at 20–22 day-old develop more lobes and less trichomes with time. TO1000 leaves were thinner with non-lobed margins. In summary, we observed that kale margins were more lobed than TO1000 (Figure 2).

### RNA-Seq and Differential Gene Expression

To identify genes that might be associated with domestication traits including leaf development in lobed kale, RNA-seq was used to assess transcriptional differences between lobed kale, cabbage, and TO1000. Three biological replicates each were sequenced; however, one of the cabbage replicates was not used for assembly because very few reads of low quality were obtained after sequencing. Between 9 and 25 million trimmed reads were obtained per sample, with ∼78–89% reads mapping uniquely to the TO1000 *B. oleracea* genome (Supplementary Table 1). After normalization with DESeq2, hierarchical clustering and principal component analysis showed that samples were grouped by morphotype, with greater variance between morphotypes than between replicates (Supplementary Figure 2).

**FIGURE 3:**
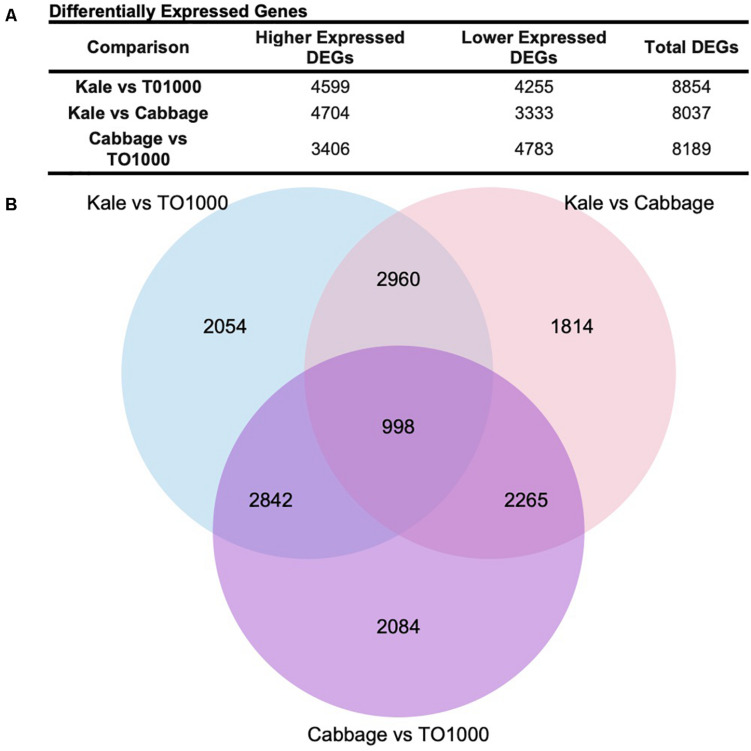
Number of genes differentially expressed in this study after comparing three different *Brassica oleracea* morphotypes (cabbage, kale, and TO1000). **(A)** Number of genes differentially expressed. **(B)** Venn Diagram showing shared genes after doing all possible comparisons between three different *B. oleracea* morphotypes.

Differentially expressed genes (DEGs) were called for all pair-wise comparisons between morphotypes (kale vs. TO1000, kale vs. cabbage, cabbage vs. TO1000), with the aim of identifying genes that were differentially expressed in kale compared to other morphotypes. Genes were considered differentially expressed if they had a twofold change in expression and a *p* < 0.05 after correction for multiple testing. In total, 8854 DEGs were identified between kale and TO1000 (4599 higher expressed in kale, 4255 lower expressed in kale), 8037 DEGs between kale and cabbage (4704 higher expressed in kale, 3333 lower expressed in kale), and 8189 DEGs between cabbage and TO1000 (3406 higher expressed in cabbage, 4783 lower expressed in cabbage) (Figure 3A). There was a total of 3958 DEGs shared between the kale–TO1000 and kale–cabbage comparisons, 2960 found only in the kale comparisons and 998 found in all three comparisons (Figure 3B).

### Polyploidy and Its Contribution to the Kale Phenotype

**FIGURE 4:**
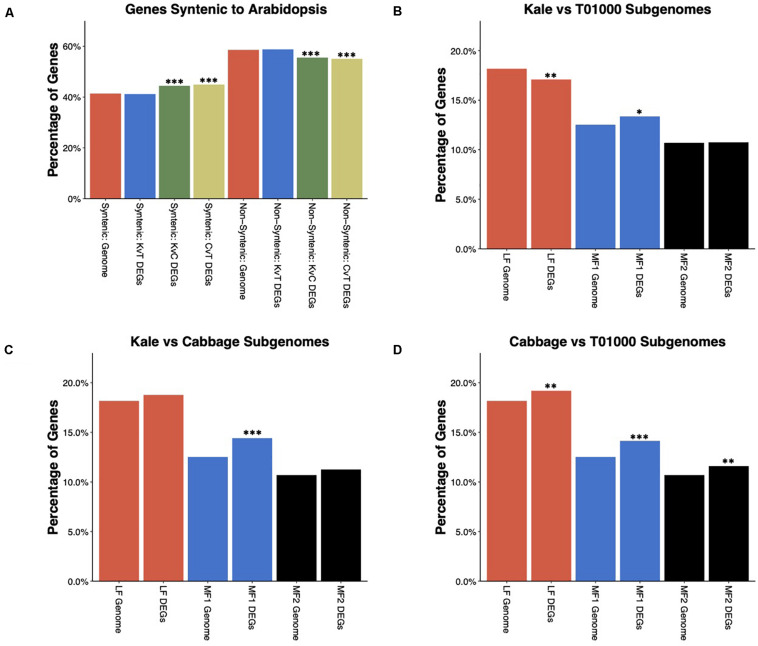
**(A)** Enrichment/depletion of differentially expressed genes (DEGs) amongst *B. oleracea* genes syntenic or non-syntenic to *Arabidopsis thaliana*. DEGs in the Kale vs TO1000 comparison show no enrichment or depletion, while the DEGs in the Kale vs Cabbage and Cabbage vs TO1000 comparisons are enriched in syntenic genes and depleted in non-syntenic genes. **(B–D)** Enrichment/depletion of DEGs for each of the three subgenomes of *B. oleracea*. **(B)** Kale vs TO1000 DEGs are depleted for LF subgenome genes, enriched for MF1 subgenome genes, and show no difference for MF2 sub genome genes. **(C)** Kale vs Cabbage DEGs are enriched for MF1 subgenome genes and show no difference for LF or MF2 subgenome genes. **(D)** Cabbage vs TO1000 DEGs show enrichment in genes from all three subgenomes. ^∗^significant at FDR corrected *p*-value < 0.05, ^∗∗^significant at FDR corrected *p*-value < 0.01, ^∗∗∗^significant at FDR corrected *p*-value < 0.001.

It has been previously shown that the whole-genome triplication (WGT) in *B. oleracea* has contributed to its phenotypic diversity (Lukens et al., 2004; Paterson, 2005). As a result, the *B. oleracea* genome is composed of three subgenomes. Previous studies have used conservation of gene order (synteny) with *A. thaliana* to assign ∼41% of annotated genes to one of the three subgenomes, which have been named the least-fractionated (LF) subgenome, more-fractionated 1 (MF1) subgenome, and more-fractionated 2 (MF2) subgenome (Parkin et al., 2014). Using these gene lists, we explored how this ancient WGT contributed to differential gene expression between morphotypes. Syntenic genes, which includes genes assigned to all three subgenomes, were significantly overrepresented, while non-syntenic genes were underrepresented among DEGs in the kale–cabbage and cabbage–TO1000 comparisons but not the kale–TO1000 comparisons (Figure 4A). We further tested the enrichment of individual subgenomes among DEGs (Figures 4C,D). The LF subgenome was underrepresented in kale–TO1000 comparison (odds ratio = 0.9162346. FDR-corrected *p*-value = 4.116097e-03) and overrepresented in the cabbage–TO1000 comparison (odds ratio = 1.0816227. FDR-corrected *p*-value = 9.948104e-03). The MF1 subgenome was significantly overrepresented for DEGs in all three comparisons (kale–TO1000: odds ratio = 1.0912796, FDR-corrected *p*-value < 1.103789e-02; kale–cabbage: odds ratio = 1.2091591, FDR-corrected *p*-value < 5.909977e-08; cabbage–TO1000: odds ratio = 1.1776233, FDR-corrected *p*-value = 2.913654e-06). The MF2 subgenome was overrepresented in the cabbage–TO1000 comparisons (odds ratio = 1.1118552. FDR-corrected *p*-value = 4.931485e-03).

### Gene Ontology and KEGG Pathway Enrichment Analysis

DEGs for each pair-wise comparison and the list of DEGs shared between the two kale comparisons (3958 genes) were separated into lists of higher- and lower-expressed genes and then tested for enrichment of Gene Ontology (GO) terms (Supplementary Tables 5–12) and Kyoto Encyclopedia of Genes and Genomes (KEGG) pathways (Supplementary Tables 13–20). We focus here specifically on those kale comparisons to better understand the kale domestication syndrome, in particular GO terms in the biological process (BP) category, as this includes developmental terms. GO terms and KEGG pathways related to the cabbage vs. TO1000 comparison are provided as supplement (Supplementary Figure 2).

#### Kale vs. TO1000

There were 91 (64 BP) and 17 (1 BP) GO terms enriched in the higher (Supplementary Table 5) and lower (Supplementary Table 6) expressed DEGs, respectively. Higher expressed genes were characterized by a number of metabolic processes: “lipid metabolism,” “nitrile metabolism,” “sulfur compound metabolism,” “benzene-containing compound metabolism,” “organic hydroxy compound biosynthesis,” and “carbohydrate derivative catabolism.” There were also multiple GO terms related to photosynthesis and plastid organization and environmental responses to both biotic and abiotic stresses. The only developmental terms enriched were related to senescence (Supplementary Figure 3A). Lower expressed genes showed little enrichment, except for genes involved in “cellular nitrogen compound metabolism” (Supplementary Figure 3B). Six KEGG pathways were enriched in higher expressed genes (Supplementary Table 13) and largely correspond to metabolic pathways identified in the GO term analysis. These include processes related to “photosynthesis-antenna proteins,” “fatty acid elongation,” “flavonoid biosynthesis,” “steroid biosynthesis,” “glyoxylate and dicarboxylate metabolism,” “other glycan degradation,” and “cyanoamino acid metabolism.” Lower expressed genes were enriched in “spliceosome” and “circadian rhythm” pathways (Supplementary Table 14).

#### Kale vs. Cabbage

Higher expressed genes had 21 (15 BP) enriched GO terms (Supplementary Table 7), while lower expressed genes (Supplementary Table 8) had 26 (11 BP). Similar to the kale vs. TO1000 comparison, there was enrichment for processes involved in “fatty acid derivative metabolism,” “nitrile metabolism,” and “photosynthesis” (Supplementary Figure 3C). There was also enrichment for environmental responses, specifically responses to herbivores. Additionally, there was enrichment for processes in “carbon fixation,” “benzoate metabolism,” “endosome organization,” and the “negative regulation of ion transport.” Lower expressed genes (Supplementary Figure 3D) include “glycosinolate metabolism,” “response to chitin,” and “gene expression.” “Sulfur compound metabolism” was enriched in both comparisons but was enriched among lower-expressed genes in the kale–cabbage comparison while being enriched in higher-expressed genes in the kale–TO1000 comparison. These are again reflected in KEGG pathways where higher expressed genes were enriched for “other glycan degradation” KEGG pathways (Supplementary Table 15). Lower expressed genes were enriched for “glucosinolate biosynthesis” and “glutathione metabolism” KEGG pathways (Supplementary Table 16).

#### Shared Genes

Higher expressed genes were enriched for 32 (22 BP) GO terms (Supplementary Table 11 and Supplementary Figure 3G). These again include multiple metabolic terms, especially related to “fatty acid derivative metabolism,” “nitrile metabolism,” “carbohydrate derivative catabolism,” and “benzoate metabolism.” Also similar to the individual comparisons, “photosynthesis” and terms related to abiotic and biotic stresses, especially herbivores, were enriched. Unique to this subgroup was an enrichment for protein phosphorylation terms. Only four GO terms were enriched in the lower expressed genes, and these were all in the cellular component GO term class (Supplementary Table 12). Higher expressed DEGs were enriched for “other glycan degradation” and “Sesquiterpenoid and triterpenoid biosynthesis” KEGG pathways, the former being also enriched in both of the individual comparisons. No KEGG pathways were enriched in the shared lower expressed DEGs (Supplementary Tables 19, 20).

### Candidate Genes Potentially Involved in the Domestication of Kale

**FIGURE 5:**
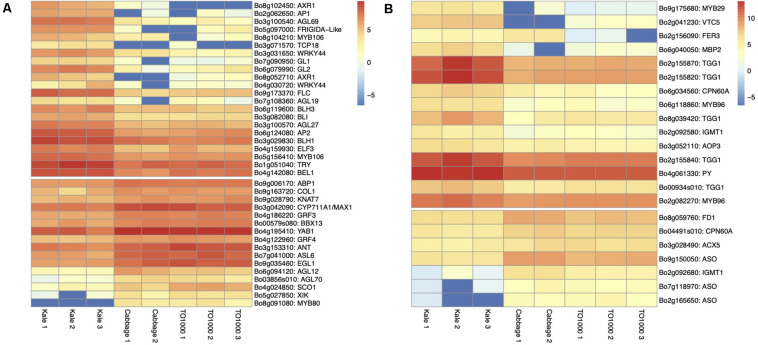
Heatmap of differentially expressed genes for lobed kale (red winter kale). Up- and downregulated genes involved in **(A)** plant and leaf development and flowering **(B)** plant defense responses and taste and nutrition.

A set of candidate genes were identified based on their expression pattern (Figure 5) and functions related to the suite of kale domestication syndrome characteristics. We further identified transcription factor (TF) families and found a total of 141 TFs in a total of 3010 differentially expressed genes (Figure 3B). Some of the most abundant included bHLH, ERF, and MYB TFs (Supplementary Table 21).

*Apical Dominance and Plant Architecture.* Several genes known to be involved in axillary shoot growth inhibition were differentially expressed in kale tissues and upregulated; these include an auxin-resistance *AXR1* (Bo8g102450, Bo8g052710) (Stirnberg et al., 1999), *TCP18* (Teosinte Branched 1-Like 1) (Bo3g071570), two copies of *REVOLUTA* (Bo9g130730, Bo7g021280) (Poza-Carrión et al., 2007), and the beta-carotene isomerase D27 chloroplastic (protein *DWARF-27* homolog) (Bo9g035190) (Waters et al., 2012). The more axillary branches gene *MAX1* (Bo3g042090) (Bennett et al., 2006) was also differentially expressed and downregulated in kale (Table 1 and Figure 5A).

**TABLE 1:** Candidate genes that are differentially expressed in lobed kale (red winter kale) or the three morphotypes.

Brassica oleracea ID	Gene ID	Possible function	Sources
**Plant development**			
***1. Apical meristem dominance***			
**Genes differentially expressed in Kale**			
Bo3g071570	*TCP18*	Regulation of secondary shoot formation in meristem buds acts downstream of MAX genes	Poza-Carrión et al., 2007
Bo9g130730 Bo7g021280	*REVOLUTA*	Epistatic to TCP18	Poza-Carrión et al., 2007
Bo3g042090	*CYP711A1/MAX1*	Synthesis and perception of mobile carotenoid signal that promotes bud arrest	Bennett et al., 2006
Bo9g035190	*DWARF27*	Acts upstream of MAX1 in the control of plant development by strigolactones	Waters et al., 2012
Bo8g102450 Bo8g052710	*AXR1*	Controls shoot branching among other activities	Stirnberg et al., 1999
**Differentially expressed genes shared by all morphotypes**			
Bo8g115290	*AXR1*	Controls shoot branching among other activities	Stirnberg et al., 1999
Bo4g182790	*PID*	Regulates auxin flow and organ formation	Bennett, 1995

***2. Leaf development***			

**Genes differentially expressed only in Kale**			
Bo7g041000	*ASL6*	Development of normal leaf shape	Semiarti et al., 2001
Bo3g039420	*NGA1*	Leaf development and regulation of leaf morphogenesis	Lee et al., 2015
Bo3g029830 Bo4g142080	*BEL1/BLH1*	Ovule development, unknown function in vegetative tissues in *B. oleracea*	Bellaoui et al., 2001
Bo4g186220	*GRF3*	Leaf development, negative regulation of cell proliferation	Beltramino et al., 2018
Bo3g082080	*BLI*	Leaf morphogenesis, positive regulation of cell division, trichome branching, and flower development	Hong et al., 2019
Bo4g122960	*GRF4*	Leaf development	Kim et al., 2003
**Differentially expressed genes shared by all morphotypes**			
Bo4g182790	*PID*	Regulates auxin flow and organ formation	Bennett, 1995
Bo5g155120	*HAT5*	Leaf margin development	Aoyama et al., 1995

***3. Trichome development***			

Bo5g156410 Bo8g104210	*MYB106*	Promote conical cell outgrowth, and in some cases, trichome initiation in diverse plant species	Gilding and Marks, 2010
Bo8g091080	*MYB80*	Epidermal cell fate specification, jasmonic acid mediated signaling pathway	Higginson et al., 2003
Bo9g035460	*EGL3*	Epidermal cell fate specification, jasmonic acid mediated signaling pathway	Morohashi et al., 2007
Bo7g090950	*GL1*	Trichome development	Morohashi et al., 2007
Bo6g079990	*GL2*	Probable transcription factor required for correct morphological development and maturation of trichomes. Regulates the frequency of trichome initiation and determines trichome spacing	Morohashi et al., 2007
Bo3g031650 Bo4g030720	*WRKY44*	Regulates trichome development, epidermal cell fate specification	Eulgem et al., 2000
Bo1g051040	*TRY*	Generation of spacing patterns among trichomes	Schnittger et al., 1999
Bo4g039530	*HDG11*	Trichome branching	Khosla et al., 2014
Bo3g162280	*CML42*	Involved in the regulation of trichome branching and herbivore defense	Dobney et al., 2009
Bo5g027850	*XIK*	Trichome branching and morphogenesis	Ojangu et al., 2012

**Flowering time**			

**Genes differentially expressed only in Kale**			
Bo3g100570	*AGL27/MAF1*	Repressor of flowering acting with FLC	Ratcliffe et al., 2001
Bo03856s010	*AGL70/MAF3*	Negative regulation of flowering acting with FLC	Ratcliffe et al., 2001
Bo3g100540	*AGL69/MAF5*	Negative regulator of flower development	Ratcliffe et al., 2001
Bo7g108360	*AGL19/*	Regulation of timing of transition from vegetative to reproductive phase	Schönrock et al., 2006
Bo6g094120	*AGL12/XAL1*	Controls flowering	Tapia-López et al., 2008
Bo9g173370 Bo3g024250	*FLC*	May inhibit flowering and inflorescence growth via a pathway involving GAI and by enhancing FLC expression and repressing FT and LFY	Madrid et al., 2020
Bo9g163720	*COL1*	Regulation of flower development	Ledger et al., 2001
Bo4g024850	*SOC1*	Negative regulation of flower development	Nasim et al., 2017
Bo4g159930	*ELF3*	Interaction between light and the circadian clock ultimately regulating flowering	Reed et al., 2000
Bo5g097000	*FRL4A*	Redundantly with MYR2 as a repressor of flowering and organ elongation under decreased light intensity	https://www.uniprot.org/uniprot/Q9LUV4-1
**Differentially expressed genes shared by all morphotypes**			
Bo4g042390	*SPL3*	Adult plants extremely dwarfed with curled up leaves, vegetative to reproductive phase transition of meristem	Cardon et al., 1997
Bo2g062650	*AP1*	Promotes flower meristem identity	Pelaz et al., 2001
Bo6g119600	*BLH3*	Regulation of timing of transition from vegetative to reproductive phase	Cole et al., 2006
Bo2g013840	*LHP1/TFL2*	Negative regulation of flowering acting with FLC	Larsson et al., 1998
Bo01054s030	*AGL68/MAF5*	Negative regulator of flower development	Ratcliffe et al., 2001
Bo3g038880	*SOC1*	Negative regulation of flower development	Nasim et al., 2017

**Defense and taste**			

**Genes differentially expressed only in Kale**			
Bo3g052110	*AOP3*	Controls the composition and amount of aliphatic glucosinolates	Wang et al., 2011
Bo9g175680	*MYB29*	Aliphatic glucosionale production	Kim et al., 2013
Bo2g092580 Bo2g092680	*IGMT1*	Indole glucosinolate metabolic process	Yi et al., 2016
Bo00934s010 Bo2g155820 Bo2g155840 Bo2g155870 Bo8g039420	*TGG1*	Degradation of glucosinolates into toxins that can deter insect herbivores	Zheng et al., 2011
Bo6g040050	*MBP2*	Myrosinase binding protein	Zheng et al., 2011
Bo3g028490	*ACX5*	Defense response to insects	Schilmiller et al., 2007
**Differentially expressed genes shared by all morphotypes**			
Bo6g095790 Bo8g039530 Bo2g155810 Bo8g063740	*TGG1*	Degradation of glucosinolates into toxins that can deter insect herbivores	Zheng et al., 2011
Bo9g094320	*IGMT4*	Indole glucosinolate metabolic process	Yi et al., 2016

**Nutrients**			

**Genes differentially expressed only in Kale**			
Bo2g041230	*VTC5*	Catalyzes a reaction of the Smirnoff–Wheeler pathway, the major route to ascorbate biosynthesis in plants	Duan et al., 2016
Bo7g118970 Bo2g165650 Bo9g150050	*ASO*	Redox system involving ascorbic acid	Fujiwara et al., 2016
Bo4g061330	*THIC*	Thiamin biosynthetic gene source of Vitamin B1	Wachter et al., 2007
Bo6g117890	*CML26*	Calcium as nutrient source	Lian et al., 2018
Bo01178s020	*CML42*	Calcium as nutrient source	Lian et al., 2018
Bo9g009850	*CP1*	Calcium as nutrient source	Jang et al., 1998
Bo4g013080	*bHLH100*	Likely regulates genes involved in the distribution of iron within the plant	Sivitz et al., 2012
Bo04491s010 Bo6g034560	RuBisCO	Plant protein source	Edelman and Colt, 2016
**Differentially expressed genes shared by all morphotypes**			
Bo3g001360	*LSC30*	Iron source	Edelman and Colt, 2016
Bo2g156090	*FER3*	Iron source	Edelman and Colt, 2016

*Leaf Development.* Differentially expressed genes in kale include two upregulated copies of *AXR1* (Bo8g052710; Bo8g102450). The lateral organ boundaries (LOB) domain-containing protein ASYMMETRIC LEAVES 2-like protein 6 (*ASL6*) (Bo7g041000) was downregulated (Semiarti et al., 2001). These genes could have a major role in the highly lobed margin kale phenotype (Figure 5A). Genes differentially expressed in all three pair-wise comparisons that were potentially involved in the kale domestication syndrome (Figure 3) include *PID* (Bo4g182790), which regulates auxin flower and organ formation (Bennett, 1995), and *HAT5* (Bo5g155120), which is involved in leaf formation and has been characterized as involved in leaf margin development (Aoyama et al., 1995).

Identified transcription factors included the AP2-like ethylene-responsive *ANT* (Bo3g153310) (Figure 5A). This gene is a positive regulator of cell proliferation, expansion, and regulation of ectopic meristems (Shigyo et al., 2006), similar to what was observed in the kale phenotypes (Figure 2Q). The growth-regulating factors 3 and 4 (*GRF3* and *GRF4*; Bo4g186220 and Bo4g122960) involved in the regulation of cell expansion in leaf and cell proliferation and the basic helix–loop–helix protein 64 *AtbHLH64* (Bo3g094060), an atypical *bHLH* transcription factor, were also downregulated in kale (Figure 5A and Supplementary Table 21).

A significant number of trichomes in kale leaves were observed during development (Figures 2N,H). More than 10 genes related to trichome formation (Table 1 and Figure 5A) were differentially expressed in kale; some of those included two upregulated copies of an Myb-related proteins *MYB106* (Bo5g156410 and Bo8g104210). These genes can act as both positive and negative regulators of cellular outgrowth and promote trichome morphogenesis and expansion (Gilding and Marks, 2010). A Homeobox-leucine zipper protein GLABRA 2-like *GL2* (Bo6g079990) was upregulated in kale. This gene is required for correct morphological development and maturation of trichomes, regulates the frequency of trichome initiation, and determines trichome spacing in *Arabidopsis* (Morohashi et al., 2007). Two expressed copies of the *WRKY* transcription factor 44 (Bo3g031650 and Bo4g030720), and the transcription factor TRIPTYCHON *TRY* (Bo1g051040) involved in trichome branching, were also differentially expressed in kale (Table 1 and Figure 5A).

*Flowering Time.* Kale phenotypes display a significant delaying in flowering time in comparison to other *B. oleracea* morphotypes, while TO1000 has been bred to be rapid cycling. We hypothesized that this maintenance in a vegetative stage was an important character during domestication due mainly to the harvest of fresh leaves for consumption. Our mature kale phenotypes did not display flowering during the course of our experiments (24 days) (Figure 1). A series of Agamous-like MADS-box proteins were exclusively expressed in kale (Figure 3B) including two downregulated genes *AGL12* (Bo6g094120) and *AGL70* (Bo03856s010) and an upregulated *AGL27* (Bo3g100570). MADS-box proteins such a *SOC1* (Bo4g024850) was downregulated, and two copies of FLOWERING LOCUS C (Bo9g173370, Bo3g024250) were upregulated (Table 1 and Figure 5A), both involved in the negative regulation of flowering (Supplementary Table 21). Other genes differentially expressed in all three pair-wise comparisons that were potentially involved in the negative regulation of flowering (Figure 3) included *SUPPRESSOR OF OVEREXPRESSION OF CO 1 SCO1* (Bo4g024850, Bo3g038880), *SPL3* (Bo4g042390), *AP1* (Bo2g062650), *BLH3* (Bo6g119600), and *AGL68/MAF5* (Bo3g100540) (Table 1 and Figure 5A).

*Plant Defense.* Differentially expressed genes involved in plant defense exclusively expressed in lobed kale leaves (Figure 3B) include two copies of the upregulated Myb-related protein *MYB96* (Bo2g082270) involved in the activation of cuticular wax biosynthesis that could potentially deter herbivores. Five upregulated copies of a glucosinolate synthesis and regulation enzyme Myrosinase *TGG1* (Bo00934s010, Bo2g155820, Bo2g155840, Bo2g155870, Bo8g039420) are involved in the degradation of glucosinolates to produce toxic degradation products that can deter insect herbivores (Zheng et al., 2011). Lastly, two copies of the indole glucosinolate O-methyltransferase, one was upregulated *IGMT1* (Bo2g092580) and the second one downregulated *IGMT1* (Bo2g092680) (Table 1 and Figure 5B), and *MYB29* (Bo9g175680) were all involved in plant defense against fungi (Supplementary Table 21). Refer to Table 1 and Figure 5B for additional candidate genes potentially involved in plant taste and defense.

*Nutritional Value of Leaves.* Kale is known to have great nutritional value including nutrients like Vitamin C, calcium, and iron (Edelman and Colt, 2016). Most notable were three copies of the L-ascorbate oxidase (*AAO*) (Ascorbase) (EC 1.10.3.3) (Bo2g165650, Bo9g150050, Bo7g118970) involved in Vitamin C synthesis, all three of which were downregulated in kale (Table 1 and Figure 5B) and the enzyme ferritin-1 *LSC30* (Bo3g001360) proposed as a source of iron. Refer to Table 1 and Figure 5B for other genes potentially involved in nutritional value of kale leaves.

## Discussion

The vegetative domestication syndrome of kale is characterized by apical dominance, leaf morphology, maintenance of a vegetative stage for high leaf production, taste/defense, and nutritional value. Differentially expressed genes (DEGs) were identified in all pair-wise comparisons. In the cabbage comparisons, DEG were enriched among syntenic genes while being depleted in non-syntenic genes (Figure 4). This aligns with recent findings that genes derived from the ancient *Brassica* polyploidy are also more likely involved in domestication in *B. rapa* (Qi et al., 2019). However, in the kale–TO1000 comparison, no such enrichment was found. As the subgenomes of *B. oleracea* have been previously reconstructed, we further explored the relative contribution of each. The LF subgenome contains the most genes and, unsurprisingly, also had the most DEGs. The LF subgenome was overrepresented in the cabbage–TO1000 comparison; however, it was underrepresented in the kale–TO1000 comparison. In contrast, the MF1 subgenome was consistently overrepresented. The more-fractionated 2 subgenome was overrepresented in the cabbage–TO1000 comparison. Given the limited number of morphotypes in this study, we cannot conclude a general pattern regarding the relative importance of syntenic vs. non-syntenic genes and each subgenome; however, the overrepresentation of the MF1 subgenome in each comparison should be explored further.

We focused our search on DEGs that were among comparisons with kale, reasoning that these were more likely to underly the unique characteristics of kale. We tested for enrichment in GO terms and KEGG pathways to gain a global view of the differences. Many more terms and pathways were enriched among higher expressed genes, despite several thousand DEGs being identified in both groups. This may suggest more coordination in the regulation of kale higher-expressed genes or simply better annotation of genes in this list. Enriched terms and pathways in all categories were typically involved in metabolism or defense. Changes in these functions would affect the taste, texture, and nutrition of leaves and thus likely have been affected by human selection. There was a relative absence of developmentally related GO terms, with the exception of senescence-related terms in the kale–TO1000 comparison. This overall lack of enrichment for developmentally related GO terms could result from either insufficient annotation of developmental genes or due to more subtle expression differences that would require tissue-specific or single-cell approaches. Alternatively, the large developmental changes observed could be caused by differential expression of only a small number of key genes. To address this, we examined sets of genes, whose function, based on homology and previous work, suggests an involvement in the developmental traits examined in this study. This analysis found many differentially expressed candidate genes shared among comparisons with kale, supporting their potential role in the kale domestication syndrome.

We identified potential candidate genes for the morphological and chemical differences among *Brassica* morphotypes. First, we found differentially expressed genes that set kale apart in terms of its morphology. Apical dominance has been proposed as a main character involved in crop domestication (Doebley et al., 1997). Suppression of axillary branches aiming to concentrate resources in the main stem is a salient phenotypic character in most *Brassica* crops. Apical dominance during domestication in kale can be explained by the indirect theory of apical dominance, as auxin-induced stem growth inhibits bud outgrowth by diverting sugars away from buds since growing stems are a strong sink for sugars (Kebrom, 2017). Here, genes involved in apical dominance and branching suppression were differentially expressed (Table 1 and Figure 5).

Kale displays morphological diversity in leaves, including leaf shape, size and margins, trichome development, colors among others, which is likely the result of genetic variation selected by plant breeders. ESEM showed that many of the characteristic leaf traits of kale are established early in leaf development. We collected tissues during kale leaf initiation and primary morphogenesis (the upper portion of the stem including immature leaves and apical and lateral meristems) to capture candidate genes related to the establishment of the basic leaf structure such as lamina initiation, specification of lamina domains, and the formation of marginal structures such as lobes or serrations.

Three types of differentially expressed genes related to the kale leaf morphogenesis phenotype were found in our samples (Table 1 and Figures 3, 5): (1) involved in margin development; (2) involved in cell proliferation, expansion, and the development of ectopic meristems; and (3) involved in trichome development. The LOB domain-containing protein 4 (asymmetric leaves 2-like protein 6) *ASL6* (Bo7g041000) was identified as a potential important gene responsible of the lobed kale leaf phenotype (Semiarti et al., 2001; Figures 1, 2E,H,K). Loss-of-function mutations in the *AS2* homolog in *Arabidopsis* have ectopic expression of class 1 *KNOX* genes in the leaves resulting in asymmetric leaf serration, with generation of leaflet-like structures from petioles and malformed veins (Matsumura et al., 2009). *ASL6* was differentially expressed and upregulated in kale. A second set of candidates involved two copies of the BEL1-like homeodomain protein 1 *BEL1/BHL1* (Bo3g029830, Bo4g142080) that were found to be overexpressed in kale. We have also found several downregulated growth-regulating factors (*GRF3* and *GRF4*) that have been shown to interact with KNOX genes (Kuijt et al., 2014). A third gene involved in cell proliferation, expansion, and the development of ectopic meristems included the NGATHA *NGA1* (Bo3g039420) involved in cell proliferation (Lee et al., 2015); however, it has been reported as downregulated for cell overproliferation and was upregulated in kale (Table 1).

In some species with simple leaves, *KNOXI* overexpression can lead to variable phenotypes, which include knot-like structures on the leaves, curled or lobed leaves, and ectopic meristems on leaves (Tsiantis and Hay, 2003). Our differential expression analysis did not show *KNOXI* overexpression; however, the differential expression of *BELL* genes, another class of TALE HD proteins found in plants, was upregulated (Bo3g029830). Yeast two-hybrid studies have indicated that *KNOX* proteins interact with BEL1-like (*BELL*) proteins. The messenger RNA (mRNA) expression patterns of *KNOX* and *BELL* proteins overlap in meristems, suggesting that they may potentially interact *in vivo* (Smith et al., 2002). Analogous *BLH–KNOX* interactions have also been reported in other plants, such as potato (*Solanum tuberosum*) and barley (*Hordeum vulgare*), indicating that these interactions are evolutionarily conserved and that the interaction is probably required for their biological function (Müller et al., 2001; Chen et al., 2003). A matter of debate is whether *KNOX* and *BLH* can exert some of their functions independently of each other or whether the formation of *KNOX/BLH* heterodimers is mandatory for TALEs to work. We did not find differential expression of *KNOX* genes in this analysis perhaps for several reasons: (1) *KNOX* do not participate in lobe formation in *B. oleracea*; (2) we did not target the correct point in development to capture *KNOX* expression in relation to leaf lobation; (3) a change in *BELL* expression is sufficient to affect *KNOX* and *BELL* interactions and alter leaf morphology without a corresponding change in *KNOX* expression. Moreover, some species, such as legumes, with dissected leaves do not express *KNOX* genes; on the contrary *KNOX* expression has been observed in leaf primordia of species with unlobed leaves, such as *Lepidium oleraceum* (Piazza et al., 2010; Vlad et al., 2014).

Epidermal cells are stimulated to differentiate into trichomes when a regulatory complex including transcription factors are triggered (Yang and Ye, 2013). ESEM results showed hairiness, and individual trichomes are seen in kale leaf blades (Figures 2N,Q). Differentially expressed genes involved in trichome formation included two *MYB* transcription factors (Higginson et al., 2003; Gilding and Marks, 2010; Pattanaik et al., 2014), the enhancer of *GL3 EGL3* (Bo9g035460), the Homeobox-leucine zipper proteins *GL1* (Bo7g090950) and *GL2* (Bo6g079990) (Morohashi et al., 2007), the transcription factor *TRY* (Bo1g051040) (Schnittger et al., 1999), and homeodomain GLABROUS 11, *HDG11* (Bo4g039530) (Khosla et a., 2014). These five genes either interact or act redundantly to promote trichome differentiation and were upregulated in kale (except *HDG11*) (Table 1 and Figure 5A). Trichome regulatory genes have been studied in *Brassica villosa*, a wild C genome relative *B. oleracea* that is densely covered by trichomes. Nayidu et al. (2014) found that *TRY* was upregulated in trichomes of *B. villosa* in contrast to *Arabidospis* and other *Brassica* species where this gene has been proposed as a negative regulator. Here, we found that *TRY* was more highly upregulated in leaves and meristems of kale in comparison to *ELG3* (Table 1), *GL1* and *GL2* (Figure 5A).

Another set of candidate genes for the kale domestication syndrome we looked for are floral transition genes. Floral transition is a major development switch controlled by regulatory pathways, integrating endogenous and environmental cues (Koornneef et al., 2004). We suggest that during kale domestication, delaying of flowering time was necessary to maintain leaf production as the main consumable. Four main regulatory pathways directly or indirectly involved in flowering time have been described in *A. thaliana*: photoperiodic, autonomous, vernalization, and gibberellin (Okazaki et al., 2007). The central integrator of flowering signals is the flowering locus C (*FLC*) that encodes a transcription factor of the MADS box family. While *A. thaliana* contains only one *FLC* gene, five copies have been described for *B. oleracea* (Okazaki et al., 2007). We found two copies of this gene being differentially expressed in kale. One was upregulated (Bo9g173370) and, together with Agamous-like MADS-box protein *AGL27/MAF1* (Bo3g100570), has been proposed as floral repressors (Ratcliffe et al., 2001; Table 1 and Figure 5A). A second copy of the *FLC* (Bo3g024250) gene was found to be shared by the three morphotypes kale, cabbage, and TO1000 (Table 1).

Pathways to domestication in kale may involve selection of plants with alterations of phytochemical concentrations (Hahn et al., 2016). Cole crops herbivore defense involves secondary metabolites (e.g., glucosinolates) and physical plant traits (e.g., waxes) that influence the feeding patterns of arthropod herbivores (Carmona et al., 2011). Glucosinolates are the main class of secondary metabolites found in cruciferous crops. They generally appear to be constitutively synthesized at rather low concentrations, but their synthesis is induced by herbivores through jasmonate and other signaling pathways. Cole crops have a two-part defense system involving glucosinolate compounds and a myrosinase protein complex. A series of copies of the Myrosinase enzyme *TGG1* were differentially expressed and upregulated in kale (Bo00934s010, Bo2g155820, Bo2g155840, Bo2g155870, Bo8g039420) (Zheng et al., 2011). This enzyme breaks down glucosinolates into toxins (isothiocyanates, thiocynates, nitriles, and epithionitriles) upon leaf tissue damage. Therefore, glucosinolates may act as a potent feeding deterrent for generalist insect species, as their toxicity causes developmental and fitness damage (Santolamazza-Carbone et al., 2014; Yi et al., 2015). Gene Ontology terms related to glucosinolates and response to herbivores were also found to be enriched in our analysis specifically in the comparison between kale vs. cabbage terms. We proposed that persistence of pungency and bitterness would have reduced competition from mammals as well as loss to pests, during cultivation and storage.

Several genes were identified to be related to the nutritional content in kale, for example, precursors and enzymes related to vitamin C; GDP-L-galactose phosphorylase 2 *VTC5* was upregulated in kale (Bo2g041230) (Duan et al., 2016), and several copies of L-ascorbate oxidase (*ASO*) (Ascorbase) (EC 1.10.3.3) and *AAO* (Bo2g165650, Bo9g150050, Bo7g118970) (Fujiwara et al., 2016) were downregulated. In addition, DEG was the precursor of the vitamin B1 phosphomethylpyrimidine synthase chloroplastic (EC 4.1.99.17) *THIC* (Bo4g061330) (Wachter et al., 2007). The RuBisCO large subunit-binding protein subunit alpha chloroplastic (60 kDa chaperonin subunit alpha (Bo04491s010) and large subunit-binding protein subunit beta chloroplastic (60 kDa chaperonin subunit beta) (Bo6g034560) were upregulated in kale (Edelman and Colt, 2016). Differentially expressed genes found in the three morphotypes included Ferredoxin-1 chloroplastic (AtFd1) *LSC30* (Bo8g059760) (Table 1 and Figure 5B).

## Conclusion

This study provided leaf developmental analysis and transcriptomics for *B. oleracea* morphotypes kale, cabbage, and TO1000, three economically important Cole crops with distinct plant morphologies and domestication syndromes. RNA-seq experiments allowed the discovery of novel candidate genes proposed to be involved in domestication syndromes. We identified DEG in *B. oleracea* that are different for each vegetative morphotypes cabbage and kale, compared to the “rapid-cycling” morphotype TO1000, and estimated the contribution of morphotype-specific gene expression patterns that set kale apart. We propose that during kale domestication, farmers could have been selecting for different vernalization times, different taste, resistance to herbivores, or flower at different times during development. The DEG we identified provides candidates for further testing the molecular basis for all of these traits.

## Data Availability Statement

The datasets presented in this study can be found in online repositories. The names of the repository/repositories and accession number(s) can be found in the article/Supplementary Material.

## Author Contributions

TA co-developed questions and framework, obtained funding, made collections, performed microscopy analysis, growth chamber experiments, and lab work including library sequencing preparation, performed analyses, and wrote manuscript. CN performed analyses, performed lab work, and wrote and edited manuscript. PM co-developed questions and framework, provided funding, and mentored students. JP co-developed questions and framework, provided funding, and mentored students. All authors contributed to the article and approved the submitted version.

## Conflict of Interest

The authors declare that the research was conducted in the absence of any commercial or financial relationships that could be construed as a potential conflict of interest.

## Footnotes

1 http://plntfdb.bio.uni-potsdam.de

2 https://github.com/niederhuth/The-molecular-basis-of-Kale-domestication

3 https://www.genome.jp/kegg/catalog/org_list.html
