# Supplementary material for: The Molecular Basis of Kale Domestication: Transcriptional Profiling of Developing Leaves Provides New Insights Into the Evolution of a Brassica oleracea Vegetative Morphotype
Source: Front Plant Sci. 2021 Mar 5;12:637115. doi: 10.3389/fpls.2021.637115 (PMC7973465; doi:10.3389/fpls.2021.637115)
Supplement: Supplementary file 1 [file Data_Sheet_1.zip › Supplementary Table 1, Figures and Table legends.pdf]

Supplementary material

Supplementary Table 1: Mapping Statistics

Sample	Total Reads	Trimmed Reads	Uniquely Mapped	Percent Uniquely Mapped
Kale 1	24535591	24516812	20190451	82.4%
Kale 2	20833408	20821507	16800807	80.7%
Kale 3	15736362	15700360	12410505	79.0%
Cabbage 1	9968261	9954978	8107108	81.4%
Cabbage 2	16648670	16624223	14775990	88.9%
TO1000 1	17083824	17002521	14426962	84.9%
TO1000 2	22918390	22886968	19362988	84.6%
TO10003	25192908	25120337	19637321	78.2%

Find Supplementary tables at : https://doi.org/10.6084/m9.figshare.13224383

Supplementary Table 2. Kale vs. TO1000 differentially expressed genes.

Supplementary Table 3. . Kale vs. Cabbage differentially expressed genes.

Supplementary Table 4. . Cabbage vs. TO1000 differentially expressed genes.

Supplementary Table 5. Kale vs TO1000 Higher Expressed Genes Enriched GO terms.

Supplementary Table 6. Kale vs TO1000 Lower Expressed Genes Enriched GO terms.

Supplementary Table 7. Kale vs Cabbage Higher Expressed Genes Enriched GO terms.

Supplementary Table 8. Kale vs Cabbage Lower Expressed Genes Enriched GO terms.

Supplementary Table 9. Cabbage vs TO1000 Higher Expressed Genes Enriched GO terms.

Supplementary Table 10. Cabbage vs TO1000 Lower Expressed Genes Enriched GO terms.

Supplementary Table 11. Kale Shared (Kale vs TO1000 & Kale vs Cabbage) Higher Expressed Genes Enriched GO terms.

Supplementary Table 12. Kale Shared (Kale vs TO1000 & Kale vs Cabbage) Lower Expressed Genes Enriched GO terms.

Supplementary Table 13. Kale vs TO1000 Higher Expressed Genes Enriched KEGG Terms.

Supplementary Table 14. Kale vs TO1000 Lower Expressed Genes Enriched KEGG Terms.

Supplementary Table 15. Kale vs Cabbage Higher Expressed Genes Enriched KEGG Terms.

Supplementary Table 16. Kale vs Cabbage Lower Expressed Genes Enriched KEGG Terms.

Supplementary Table 17. Cabbage vs TO1000 Higher Expressed Genes Enriched KEGG Terms.

Supplementary Table 18. Cabbage vs TO1000 Lower Expressed Genes Enriched KEGG Terms.

Supplementary Table 19. Kale Shared (Kale vs TO1000 & Kale vs Cabbage) Higher Expressed Genes Enriched KEGG Terms.

Supplementary Table 20. Kale Shared (Kale vs TO1000 & Kale vs Cabbage) Lower Expressed Genes Enriched KEGG Terms.

Supplementary Table 21. Enriched Transcription Factor list for transcription factors that were exclusively expressed in kale or shared among the three morphotypes

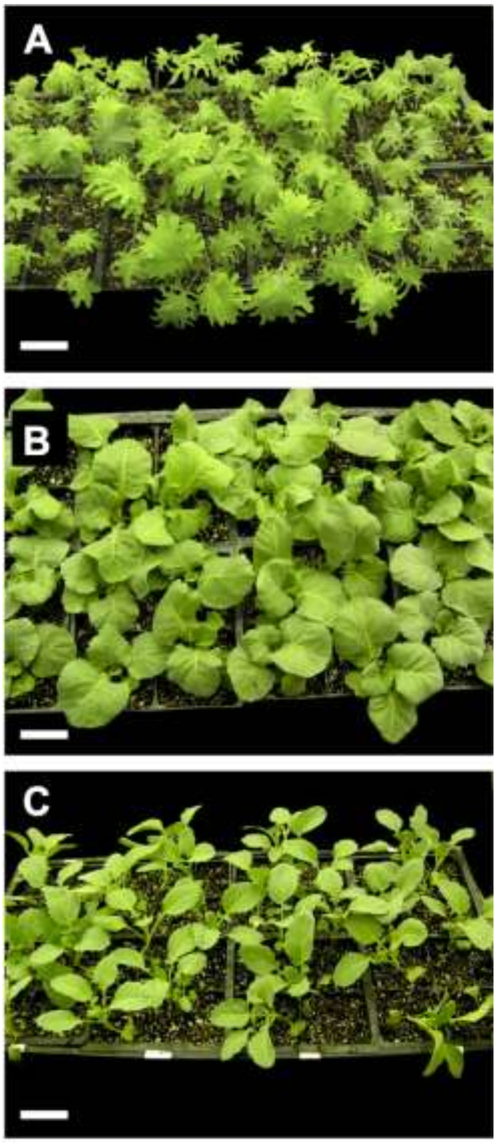

Supplementary Figure 1. Time point (24 days after planted)at which is morphotype was collecting during the RNAseq experiments. (A) red winter kale (*Brassica oleraceae* var. acephala *Red Winter*) with three to four fully expanded leaves. (B) cabbage PI 303629 South Africa (*B. oleracea* var. *capitate* with two to three fully expanded leaves. (C) TO1000 (*B. oleracea* var. *alboglabra*) three to five fully expanded leaves picture provided by Zachary Stansell. Scale bar = 10 cm

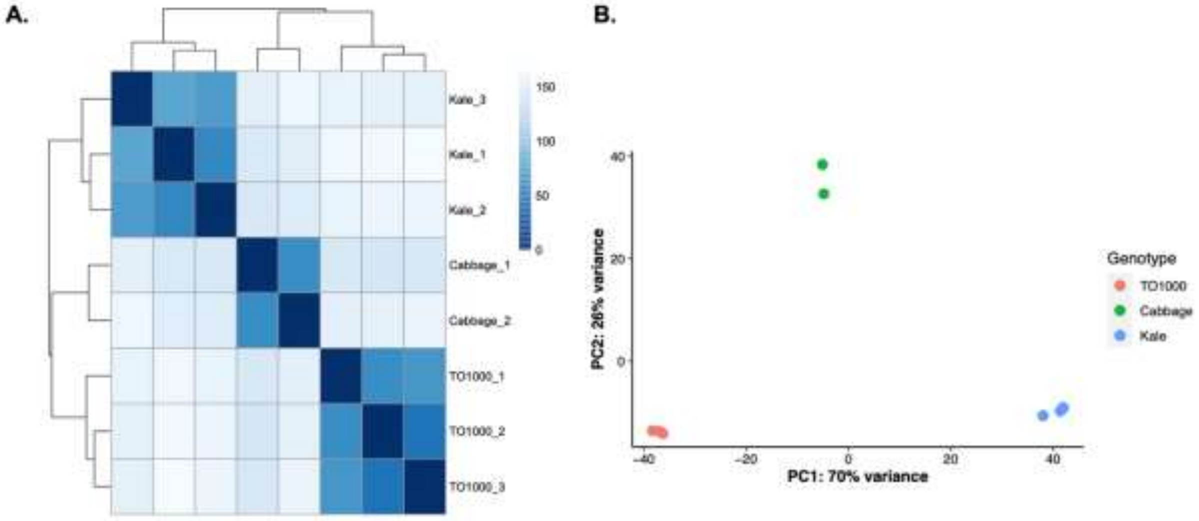

Supplementary Figure 2. Comparative summary statistics of the reference based assembly and data quality for each sample. A: Heat plot showing data quality and stronger correlations among samples belonging to the same morphotype. B: Percentage of variance among each condition and replicates.

A.

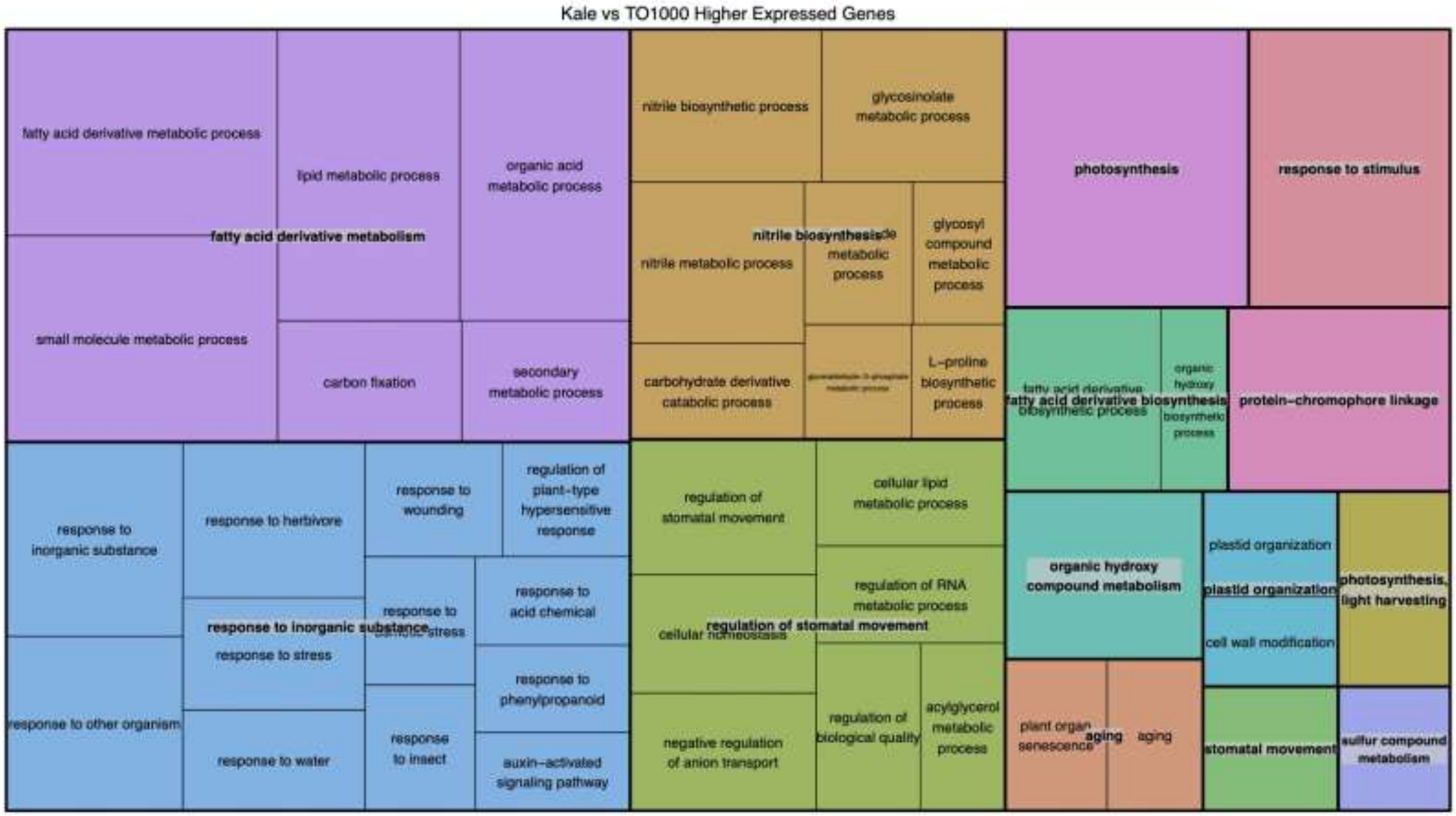
Kale vs TO1000 Higher Expressed Genes
fatty acid derivative metabolic process
lipid metabolic process
organic acid metabolic process
fatty acid derivative metabolism
small molecule metabolic process
carbon fixation
secondary metabolic process
nitrile biosynthetic process
glycosinolate metabolic process
nitrile biosynthesis
metabolic process
glycosyl compound metabolic process
nitrile metabolic process
carbohydrate derivative catabolic process
L-proline biosynthetic process
photosynthesis
response to stimulus
fatty acid derivative biosynthetic process
fatty acid derivative biosynthesis
organic hydroxy biosynthetic process
protein-chromophore linkage
response to inorganic substance
response to herbivore
response to wounding
regulation of plant-type hypersensitive response
response to acid chemical
response to inorganic substance
response to stress
response to osmotic stress
response to phenylpropanoid
response to other organism
response to water
response to insect
auxin-activated signaling pathway
regulation of stomatal movement
cellular lipid metabolic process
regulation of RNA metabolic process
cellular homeostasis
regulation of stomatal movement
negative regulation of anion transport
regulation of biological quality
acylglycerol metabolic process
organic hydroxy compound metabolism
plastid organization
plastid organization
cell wall modification
photosynthesis, light harvesting
plant organ senescence
aging
aging
stomatal movement
sulfur compound metabolism

B.

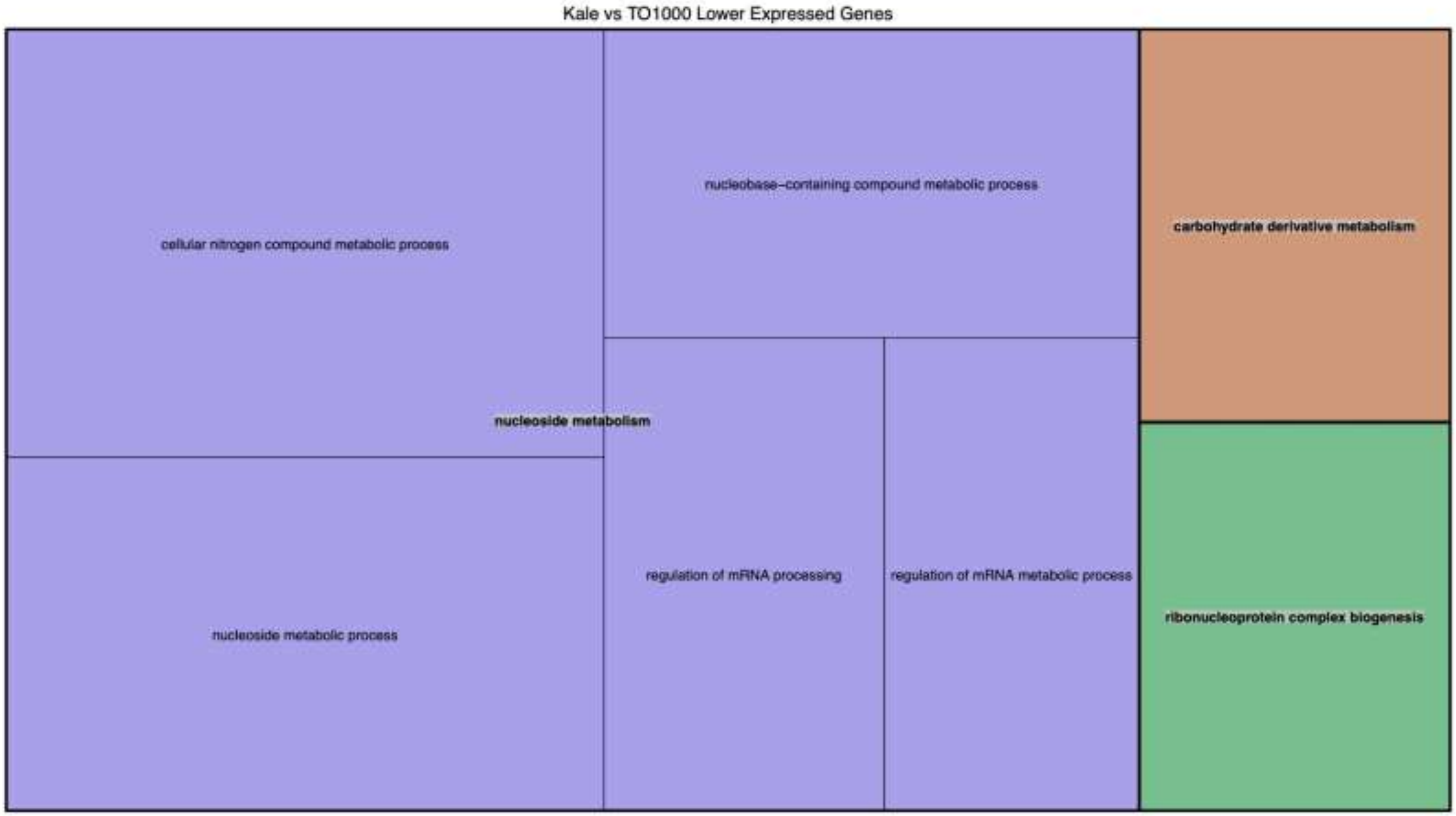
Kale vs TO1000 Lower Expressed Genes
cellular nitrogen compound metabolic process
nucleobase−containing compound metabolic process
carbohydrate derivative metabolism
nucleoside metabolism
regulation of mRNA processing
regulation of mRNA metabolic process
ribonucleoprotein complex biogenesis
nucleoside metabolic process

C.

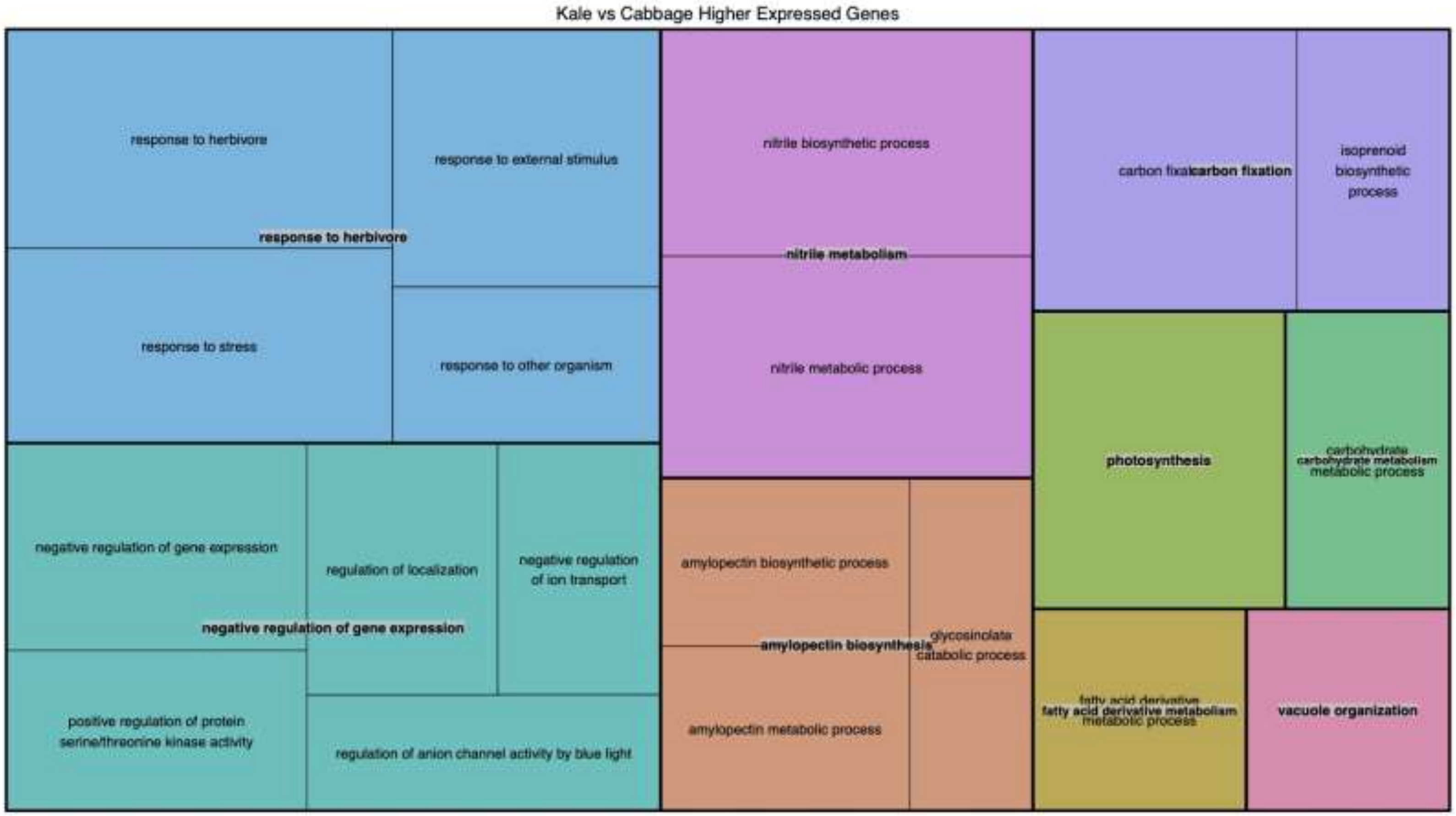
Kale vs Cabbage Higher Expressed Genes
response to herbivore
response to external stimulus
response to herbivore
response to stress
response to other organism
nitrile biosynthetic process
nitrile metabolism
nitrile metabolic process
carbon fixation
carbon fixation
isoprenoid biosynthetic process
photosynthesis
carbohydrate metabolic process
carbohydrate metabolism
negative regulation of gene expression
regulation of localization
negative regulation of ion transport
negative regulation of gene expression
positive regulation of protein serine/threonine kinase activity
regulation of anion channel activity by blue light
amylopectin biosynthetic process
amylopectin biosynthesis
glycosinolate catabolic process
amylopectin metabolic process
fatty acid derivative metabolic process
fatty acid derivative metabolism
vacuole organization

D.

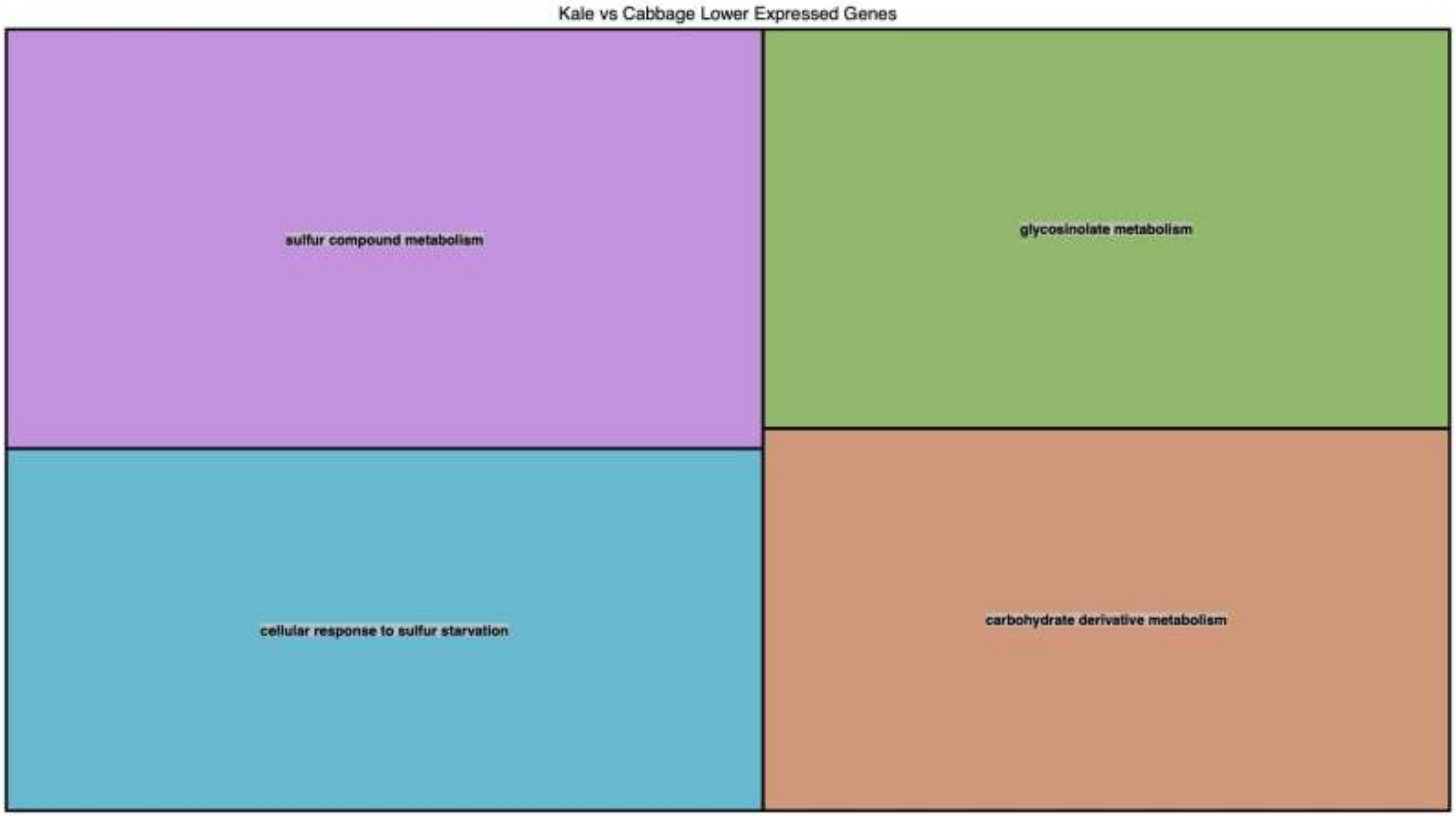
Kale vs Cabbage Lower Expressed Genes
sulfur compound metabolism
glycosinolate metabolism
cellular response to sulfur starvation
carbohydrate derivative metabolism

E.

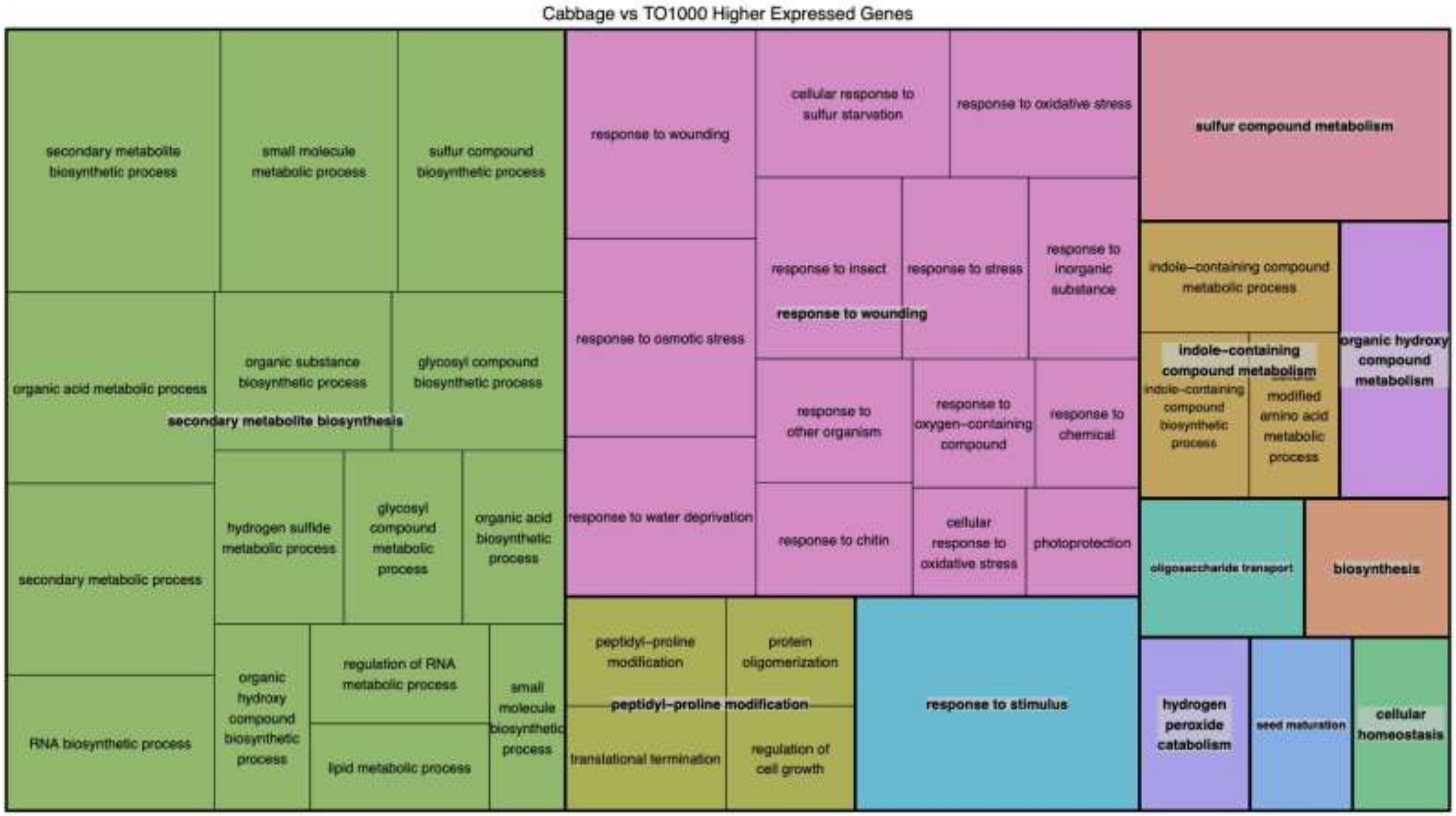

Cabbage vs TO1000 Higher Expressed Genes
secondary metabolite biosynthetic process
small molecule metabolic process
sulfur compound biosynthetic process
organic acid metabolic process
organic substance biosynthetic process
glycosyl compound biosynthetic process
secondary metabolite biosynthesis
secondary metabolic process
hydrogen sulfide metabolic process
glycosyl compound metabolic process
organic acid biosynthetic process
RNA biosynthetic process
organic hydroxy compound biosynthetic process
regulation of RNA metabolic process
lipid metabolic process
small molecule biosynthetic process
response to wounding
cellular response to sulfur starvation
response to oxidative stress
response to osmotic stress
response to insect
response to stress
response to inorganic substance
response to wounding
response to other organism
response to oxygen-containing compound
response to chemical
response to water deprivation
response to chitin
cellular response to oxidative stress
photoprotection
peptidyl-proline modification
protein oligomerization
peptidyl-proline modification
translational termination
regulation of cell growth
response to stimulus
sulfur compound metabolism
indole-containing compound metabolic process
indole-containing compound metabolism
indole-containing compound biosynthetic process
modified amino acid metabolic process
organic hydroxy compound metabolism
oligosaccharide transport
biosynthesis
hydrogen peroxide catabolism
seed maturation
cellular homeostasis

F.

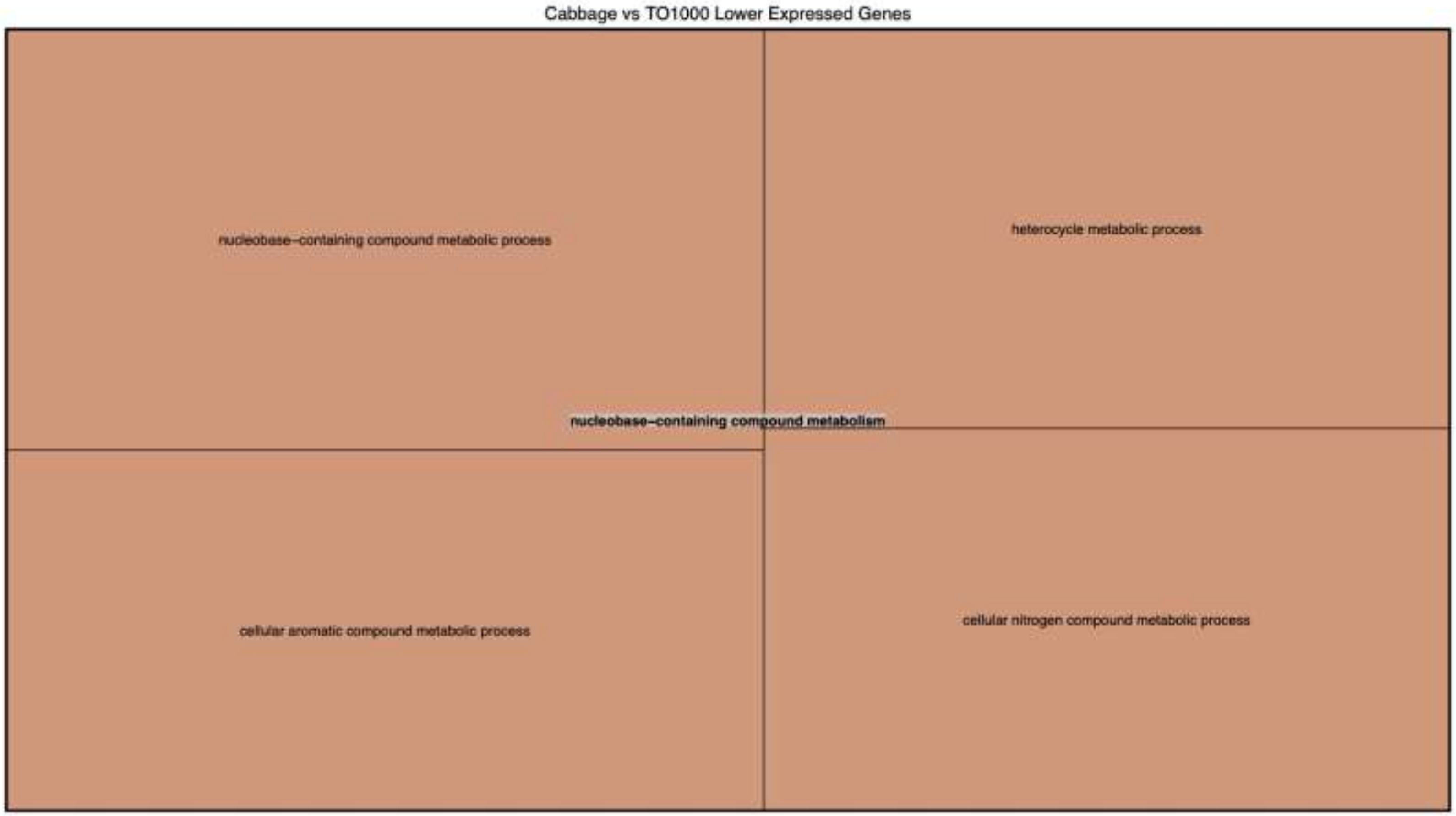
Cabbage vs TO1000 Lower Expressed Genes
nucleobase−containing compound metabolic process
heterocycle metabolic process
nucleobase−containing compound metabolism
cellular aromatic compound metabolic process
cellular nitrogen compound metabolic process

G.

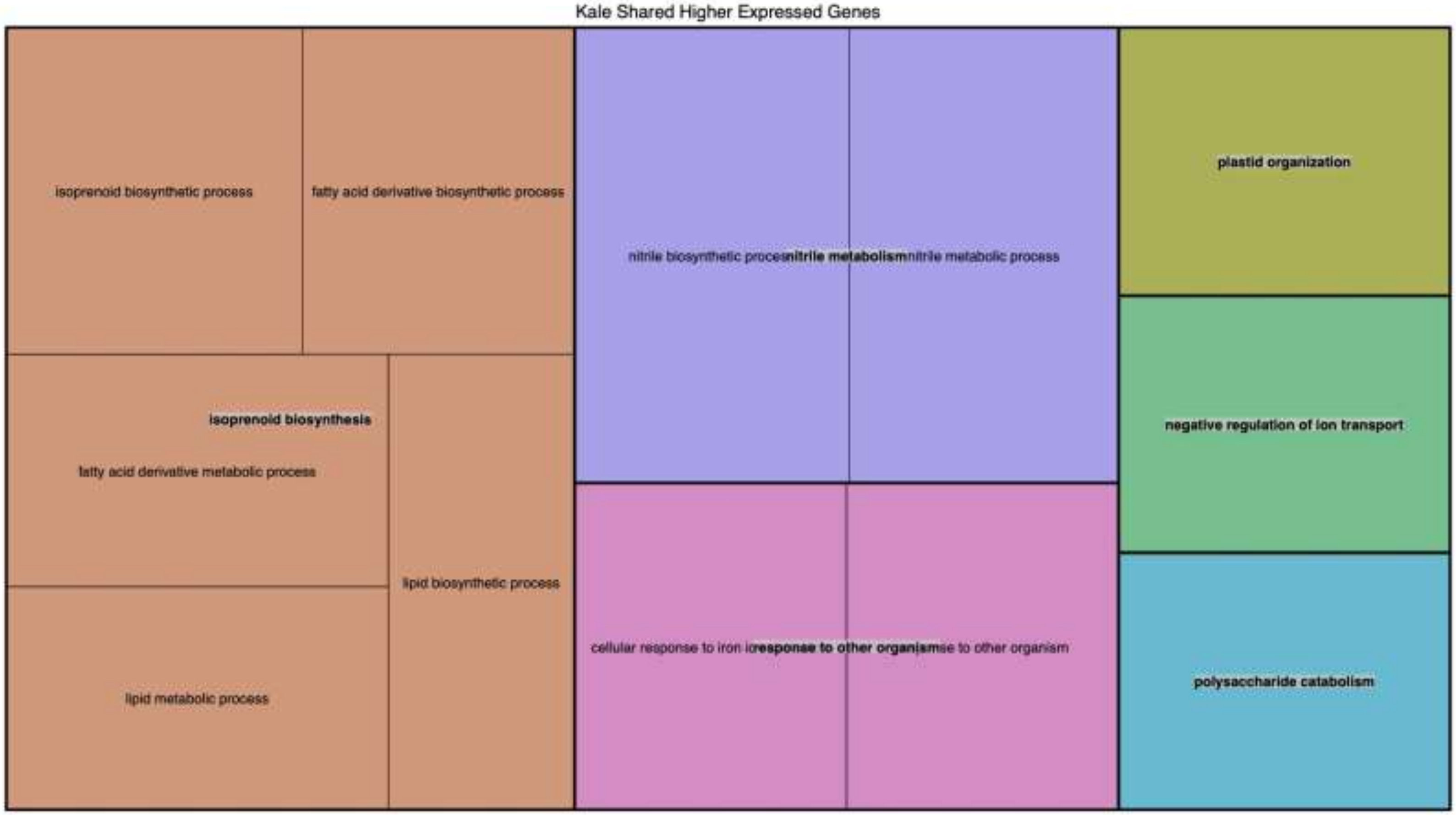
Kale Shared Higher Expressed Genes
isoprenoid biosynthetic process
fatty acid derivative biosynthetic process
isoprenoid biosynthesis
fatty acid derivative metabolic process
lipid biosynthetic process
lipid metabolic process
nitrile biosynthetic proces nitrile metabolism nitrile metabolic process
cellular response to iron io response to other organism se to other organism
plastid organization
negative regulation of ion transport
polysaccharide catabolism

Supplementary figure 3. REVIGO treemaps summarizing gene ontology categories over-represented in kale leaves and meristems tissues. For comparisons between TO1000, cabbage and kale and separating lower and higher expressed genes. A. Kale vs TO100 higher expressed genes; B. Kale vs TO1000 lower expressed genes; C. Kale vs cabbage higher expressed genes; D. Kale vs cabbage lower expressed genes E. Cabbage vs TO1000 higher expressed genes F. Cabbage vs TO1000 lower expressed genes G. Kale shared higher expressed genes

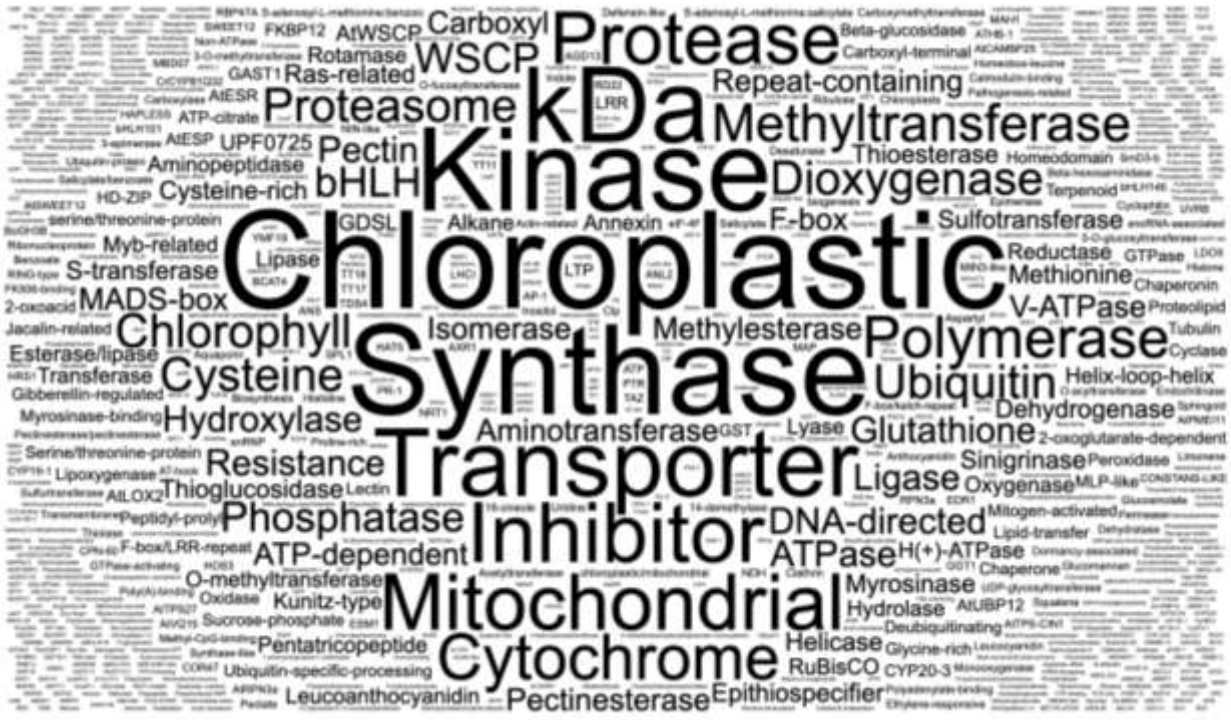

Supplementary Figure 4. Word cloud for differentially expressed genes unique to Kale among leaf and meristematic tissues of *Brassica oleracea* morphotypes, emphasizing the predominant role of chloroplast and mitochondrial genes, Kinases, Proteases and Polymerases, also genes involved in transporting substances and inhibiting reactions (see Fig. 2B). A complete list of enriched terms can be found in Suppl. Table 21.
